# E-DES-PROT: A novel computational model to describe the effects of amino acids and protein on postprandial glucose and insulin dynamics in humans

**DOI:** 10.1016/j.isci.2023.106218

**Published:** 2023-02-18

**Authors:** Bart van Sloun, Gijs H. Goossens, Balázs Erdõs, Shauna D. O’Donovan, Cécile M. Singh-Povel, Jan M.W. Geurts, Natal A.W. van Riel, Ilja C.W. Arts

**Affiliations:** 1 TiFN, Wageningen, the Netherlands; 2 Maastricht Centre for Systems Biology (MaCSBio), Maastricht University, Maastricht, the Netherlands; 3 Department of Human Biology, NUTRIM School of Nutrition and Translational Research in Metabolism, Maastricht University, Maastricht, the Netherlands; 4 Division of Human Nutrition and Health, Wageningen University, Wageningen, the Netherlands; 5 Department of Biomedical Engineering, Eindhoven University of Technology, Eindhoven, the Netherlands; 6 FrieslandCampina, Amersfoort, the Netherlands; 7 Department of Experimental Vascular Medicine, Amsterdam University Medical Centers, location AMC, Amsterdam, the Netherlands

**Keywords:** Biomolecules, Human metabolism, In silico biology

## Abstract

Current computational models of whole-body glucose homeostasis describe physiological processes by which insulin regulates circulating glucose concentrations. While these models perform well in response to oral glucose challenges, interaction with other nutrients that impact postprandial glucose metabolism, such as amino acids (AAs), is not considered. Here, we developed a computational model of the human glucose-insulin system, which incorporates the effects of AAs on insulin secretion and hepatic glucose production. This model was applied to postprandial glucose and insulin time-series data following different AA challenges (with and without co-ingestion of glucose), dried milk protein ingredients, and dairy products. Our findings demonstrate that this model allows accurate description of postprandial glucose and insulin dynamics and provides insight into the physiological processes underlying meal responses. This model may facilitate the development of computational models that describe glucose homeostasis following the intake of multiple macronutrients, while capturing relevant features of an individual’s metabolic health.

## Highlights

• We developed a computational model that incorporates postprandial effects of AAs
• Introducing AAs is an important move toward describing complex meals
• E-DES-PROT is able to accurately describe postprandial glucose and insulin dynamics
• The model allows insight into physiological processes underlying meal responses

## Graphical abstract

**Figure:**
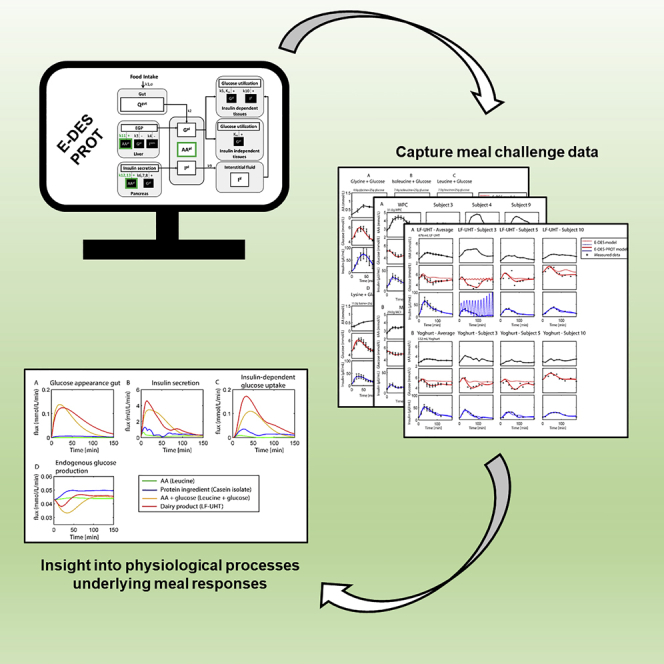

## Introduction

Glucose homeostasis is primarily regulated by the hormones insulin and glucagon, which act in antagonistic fashion to maintain circulating glucose concentrations within a healthy range.^1^^,^^2^ When glucose concentrations are elevated (i.e. following meal intake), pancreatic β*-*cells secrete insulin to suppress hepatic glucose output and promote glucose uptake in peripheral organs, predominantly in the skeletal muscle.^3^ In contrast, when glucose concentrations drop (i.e. during fasting or physical exercise), pancreatic α-cells secrete glucagon to stimulate glycogen breakdown and gluconeogenesis (formation of glucose from non-carbohydrate precursors), allowing glucose release from the liver into the circulation, thereby preventing hypoglycemia.^4^ As such, glucagon and insulin exert opposing actions on glucose metabolism and are part of a tightly regulated feedback system to maintain glucose homeostasis.

Computational models of whole-body glucose homeostasis describe and incorporate the current mechanistic understanding of insulin-mediated regulation of circulating glucose concentrations.^5^^,^^6^^,^^7^ These processes are represented by model parameters, which can be estimated from postprandial time-series data without requiring direct invasive measurements. One of the earliest computational glucose models, the Bergman minimal model,^5^ was able to determine *insulin sensitivity* (i.e. the capability of insulin to suppress hepatic glucose output and increase glucose disposal in insulin-sensitive tissues) and *glucose effectiveness* (i.e. the ability of glucose to enhance its own disposal at basal insulin levels) in response to an intravenous glucose tolerance test. The Bergman minimal model formed the basis of the Food and Drug Administration–approved glucose-insulin model by Dalla Man and colleagues,^6^^,^^8^ which is used for *in silico* simulation and testing of insulin pump systems. The Dalla Man model has been parameterized using triple tracer glucose data to allow quantification of glucose fluxes between tissues.

The Eindhoven-Diabetes Education Simulator (E-DES), a multi-compartmental ordinary differential equation model, has been used to describe glucose dynamics following a glucose challenge in healthy individuals as well as patients with type 1 and type 2 diabetes.^7^^,^^9^^,^^10^ We have previously individualized the E-DES model to allow accurate description of individual postprandial responses compared to population-based models, demonstrating it is capable of providing mechanistic insight into glucose homeostasis of individuals.^11^ While the E-DES model performs very well in response to an oral glucose challenge, modeling the response to more complex meals is still challenging because these contain fat and protein, which also influence glucose homeostasis.

Dietary protein consists of amino acids (AAs) which are used for synthesis of body protein and of nitrogen-containing compounds, such as creatine, peptide hormones, and several neurotransmitters.^12^ AAs have been shown to influence glucose metabolism by inducing insulin secretion to facilitate AA uptake and incorporation into protein in muscle tissue, and secreting glucagon to enhance hepatic AA uptake, production of ketone bodies from AAs, and formation of glucose from AAs (i.e. gluconeogenesis).^2^^,^^4^ In a systematic review, we have recently summarized available studies describing postprandial glucose and insulin responses to AAs.^13^

In the present study, we aimed to extend an existing computational model of the glucose-insulin regulatory system to account for the postprandial effects of AAs. To parameterize the model, we used time-series data of postprandial AA, glucose and insulin concentrations following AA challenges (with and without glucose), dried milk protein ingredients, and dairy products, derived both from a previously performed randomized, single-blind crossover trial^14^ as well as data extracted from available literature.^13^ Here, we show that this novel model, which we termed E-DES-PROT, accurately describes postprandial glucose and insulin dynamics, outperforms the original E-DES model, and allows insight into the physiological processes underlying meal responses.

## Results

### Postprandial simulation of AA, glucose, and insulin dynamics following AA challenges and intake of protein ingredients

We investigated whether our newly developed model was able to capture AA and protein challenges, estimating only the model parameters accounting for AAs (*k*11*-k*13). The parameters pertaining to the original E-DES model were kept to their healthy average population value and the measured plasma AA concentration (pertaining to the challenge) was interpolated and provided to the model as an input.^9^

**Figure 1:**
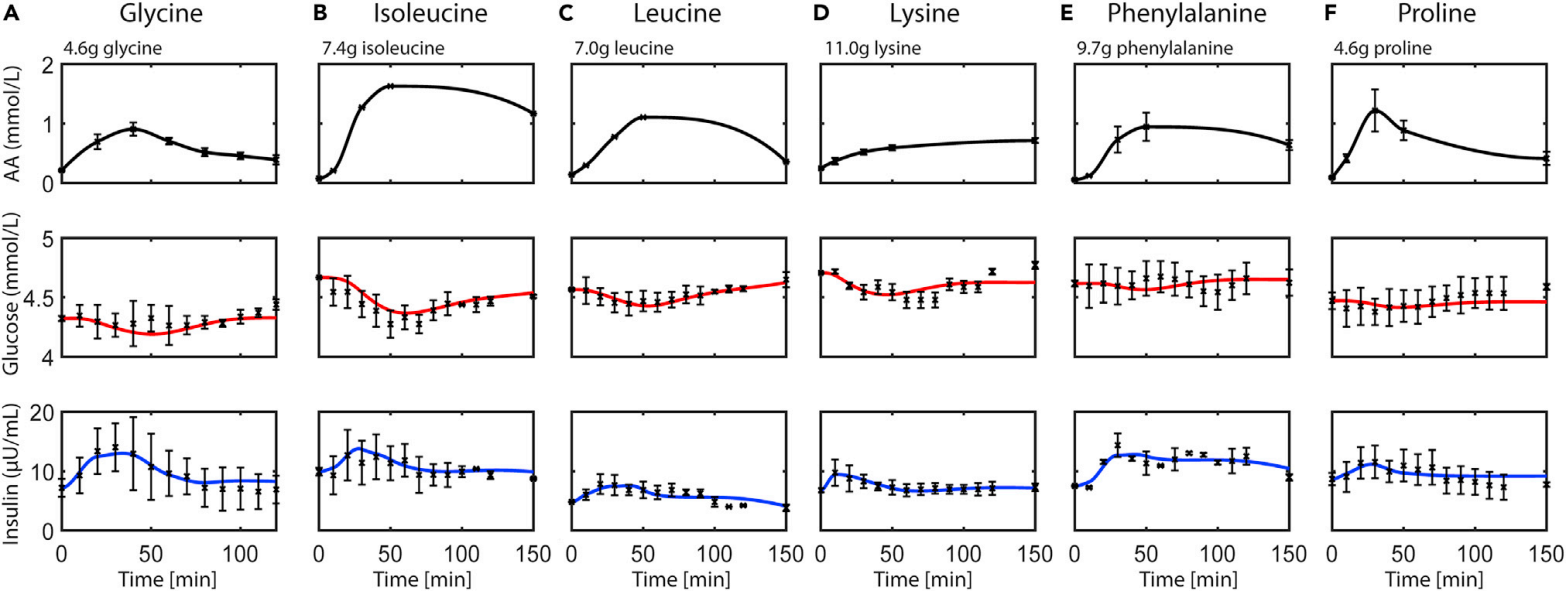
Simulated postprandial responses in the literature study following ingestion of amino acids The model parameters pertaining to amino acids (AAs, *k*11–*k*13) were estimated, whereas the other parameters were kept to their original population value. The tAA input is shown in black (data and polynomial interpolation). The simulated glucose and insulin concentrations are shown in red and blue, respectively. The measured concentrations, obtained from van Sloun et al.,^13^ are shown as black asterisk with corresponding standard errors of the means.

**Figure 2:**
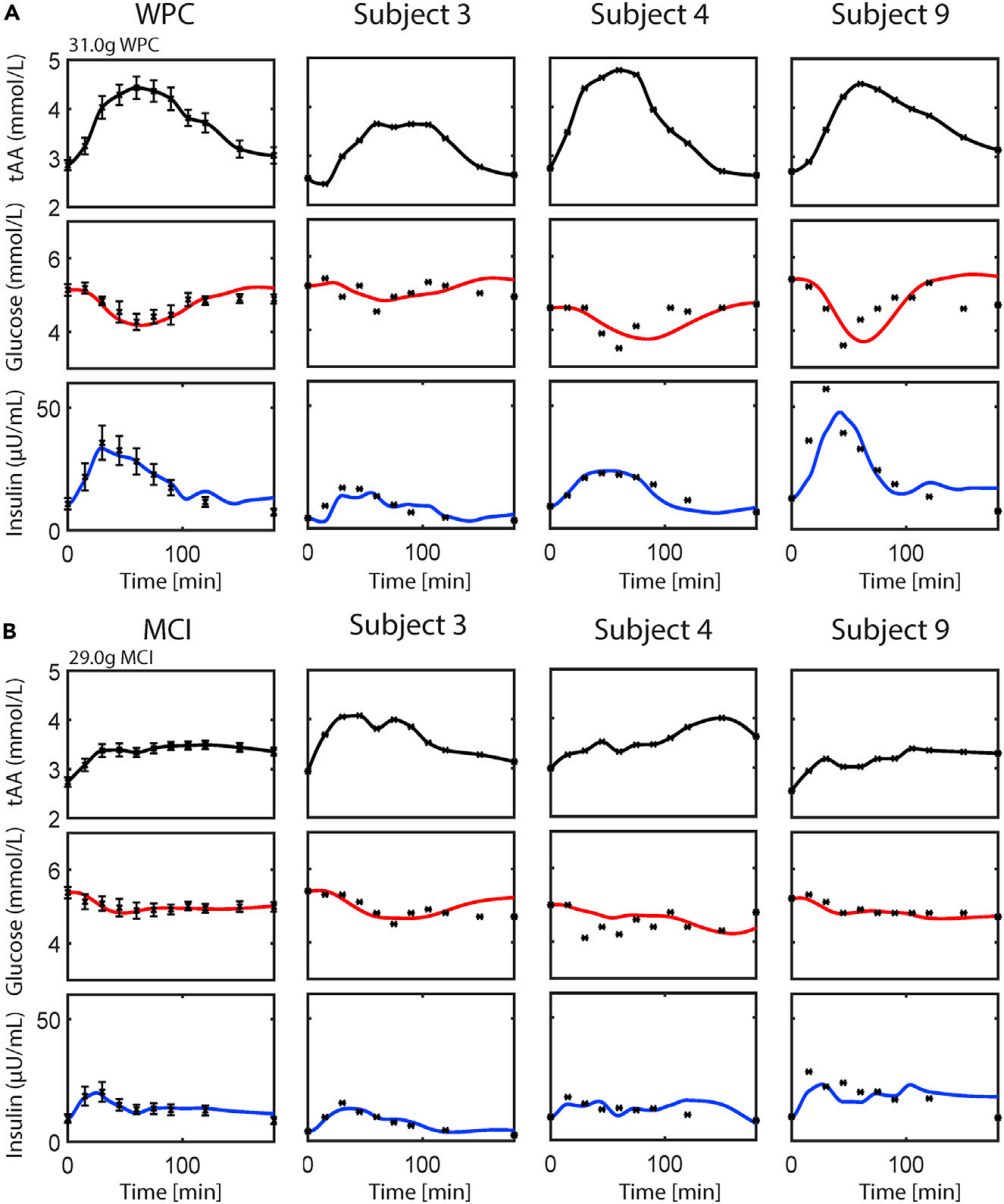
Plasma glucose and insulin simulation following intake of whey protein concentrate (WPC) and micellar casein isolate (MCI) in the average healthy study population and selected individuals The model parameters pertaining to amino acids (AAs, *k*11–*k*13) were estimated, whereas the other model parameters were kept to their original population value. The tAA input is shown in black (data and polynomial interpolation). The simulated glucose and insulin concentrations are shown in red and blue, respectively. The measured concentrations, obtained from Horstman et al.*,*^14^ are shown as black asterisks with corresponding standard errors of the means. The leftmost column in panel (A and B) pertains to average study population, whereas the other columns represent selected individuals.

The simulated glucose and insulin responses, parameterized on the AA challenges (1 mmol/kg body weight), are shown in Figure 1. The simulated glucose and insulin responses, parameterized on the milk protein ingredients (i.e. WPC and MCI) containing 25 g of protein in a 700 mL solution, are shown in Figure 2. Here, the leftmost column pertains to the average population responses, whereas the other columns show selected individual responses highlighting striking model behavior. The complete overview of all the individual glucose and insulin responses is shown in Data S2.

Visual inspection of the plasma glucose and insulin simulations following the AA challenges and protein ingredients displays good agreement with the measured data. In general, our new model is able to capture the postprandial glucose and insulin following AA challenges, as well as protein ingredients. In addition, our model is also able to capture individual glucose and insulin concentrations following the intake of protein ingredients, being able to capture more pronounced glucose and insulin responses (Figure 2A, subject 9), but also less prominent responses (Figure 2A, subject 3).

### E-DES-PROT improves upon the original E-DES model in capturing glucose dynamics following the intake of AA + glucose and dairy products

We investigated whether our newly developed model was able to capture meals that in addition to AAs and protein also contained glucose and carbohydrates. The E-DES-PROT model was compared to the original E-DES model using the AIC and BIC, with the lowest AIC and BIC value pertaining to the preferred model.

### Amino acids + glucose challenge

**Figure 3:**
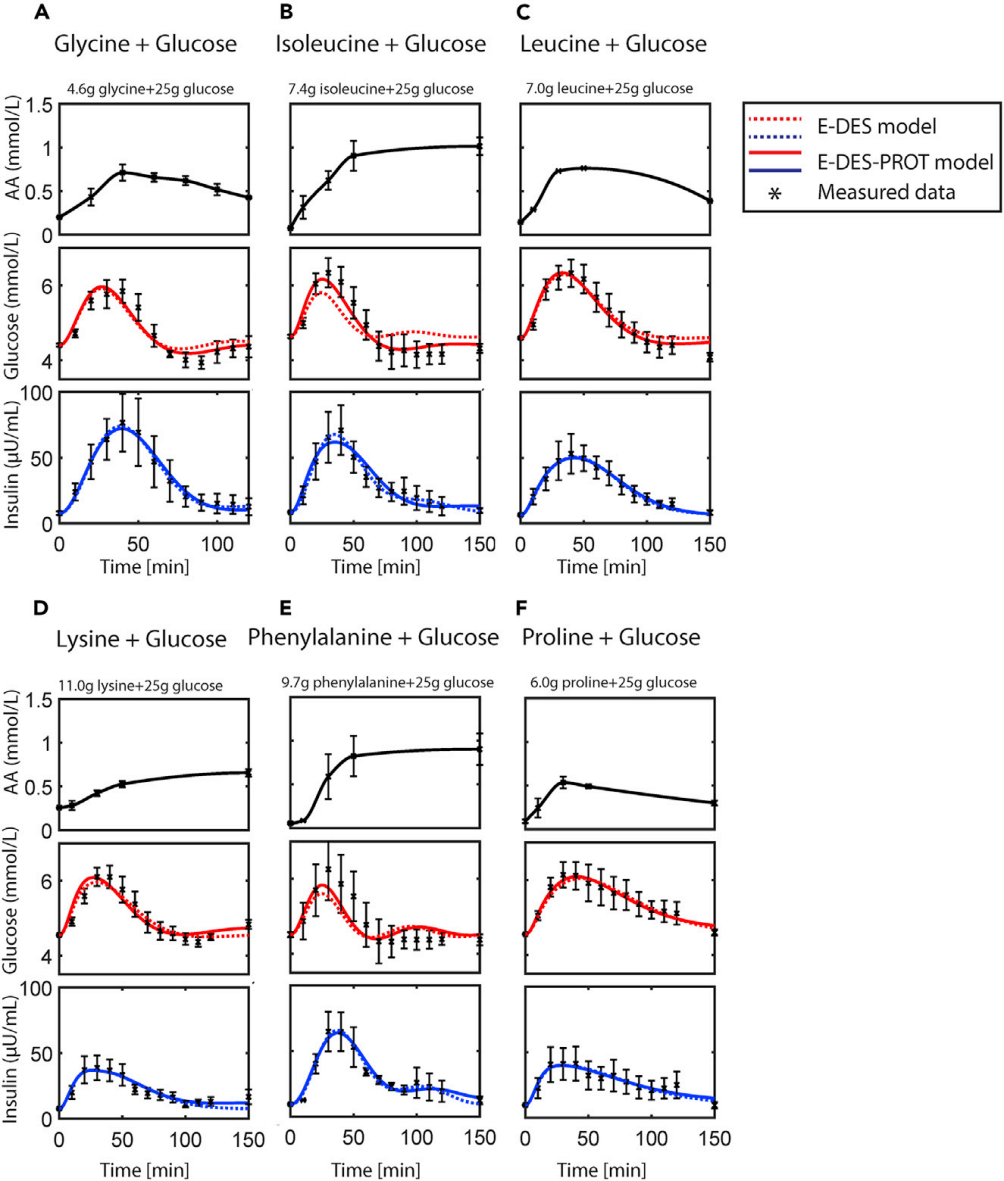
Plasma glucose and insulin simulation following intake of different amino acids (AAs) together with glucose in healthy individuals, using the original E-DES and E-DES-PROT model The AA input is shown in black (data and polynomial interpolation). The simulated glucose and insulin concentrations following parameter estimation (*k*1, *k*5, *k*6, and *k*8) using the original E-DES model, are shown in dashed red and blue, respectively. The simulated glucose and insulin concentrations following parameter estimation (*k*1, *k*5, *k*6, *k*8, and *k*11–*k*13) using the E-DES-PROT model, are shown in red and blue, respectively. The other model parameters were kept to their original population value. The measured concentrations, obtained from Sloun et al.,^13^ are shown as black asterisks with corresponding standard errors of the means.

The simulated glucose and insulin responses using the original E-DES and the newly developed E-DES-PROT model, parameterized on the AA + glucose challenges (1 mmol/kg body weight +25 g glucose), are shown in Figure 3. For the original E-DES model, parameters (*k*1, *k*5, *k*6, and *k*8) were estimated. For the E-DES-PROT model, these model parameters were estimated in conjunction with the model parameters accounting for AAs (*k*11–*k*13). The measured plasma AA concentration (pertaining to the challenge) was interpolated and provided to the model as an input.^9^

Visual inspection of the plasma glucose and insulin simulations following the AA + glucose challenges displays good agreement with the measured data using the E-DES and E-DES-PROT model. The E-DES-PROT model is able to capture AA + glucose challenges and improves in capturing the measured postprandial glucose data (Figure 3, AIC, 1.05 and −1.45; BIC, 3.31 and 2.50 for E-DES, and E-DES-PROT, respectively, across all challenges). For glycine + glucose (Figure 3A), the improvement pertained to the period from 60 min after intake onward, whereas the E-DES-PROT model improved the overall postprandial glucose response for isoleucine + glucose (Figure 3B). The postprandial insulin data were nicely captured using both models. Thus, both the E-DES and E-DES-PROT model are able to describe postprandial responses to simple meal challenges consisting of single AAs co-ingested with glucose. The complete overview of the AIC and BIC for the AA + glucose challenges using the E-DES and E-DES-PROT model is shown in Table S1.

### Dairy products

**Figure 4:**
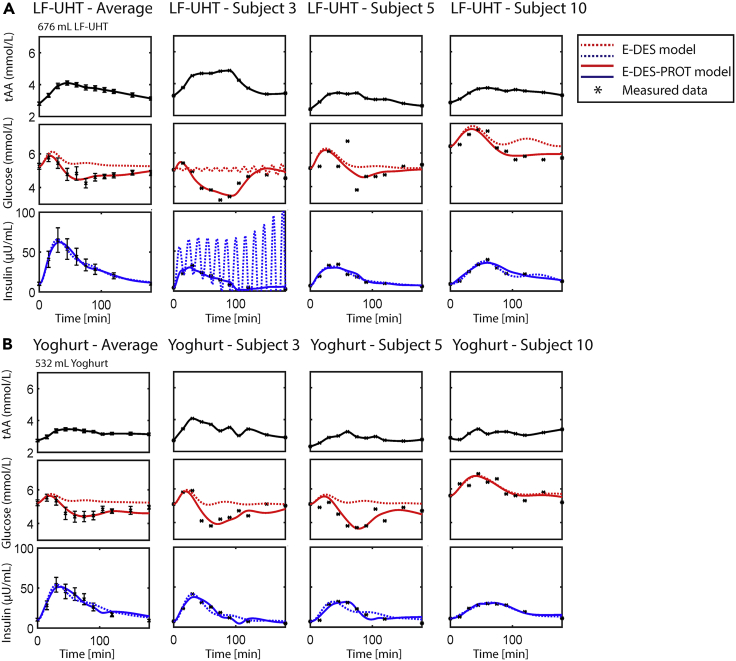
Plasma glucose and insulin simulation following intake of low-fat untreated treated milk (LF-UHT) and yoghurt in the average healthy study population and selected individuals using the original E-DES and E-DES-PROT model The tAA input is shown in black (data and polynomial interpolation). The simulated glucose and insulin concentrations following parameter estimation (*k*1, *k*5, *k*6, and *k*8) using the original E-DES model, are shown in dashed red and blue, respectively. The simulated glucose and insulin concentrations following parameter estimation (*k*1, *k*5, *k*6, *k*8, and *k*11–*k*13) using the E-DES-PROT model, are shown in red and blue, respectively. The other model parameters were kept to their original population value. The measured concentrations, obtained from Horstman et al.,^14^ are shown as black asterisks with corresponding standard errors of the means. The leftmost column in panel A & B pertains to average study population, whereas the other columns represent selected individuals.

The simulated glucose and insulin responses using the original E-DES and the newly developed E-DES-PROT model, parameterized on responses to selected dairy food products (i.e. low-fat untreated milk (LF-UHT) and yoghurt) containing 25 g of protein and a variable amount of carbohydrates in a 700 mL solution, are shown in Figure 4. Here, the leftmost column pertains to the average population responses, whereas the other columns show selected individual responses highlighting striking model behavior. The complete overview of the individual glucose and insulin responses for the dairy products (i.e. LF-UHT, LF-PAS, FF-UHT, FF-PAS, and yoghurt) is shown in Data S3. For the original E-DES model, parameters (*k*1, *k*5, *k*6, and *k*8) were estimated. For the E-DES-PROT model, these parameters were estimated in conjunction with the model parameters accounting for AAs (*k*11–*k*13). The measured plasma AA concentration (pertaining to the challenge) was interpolated and provided to the model as an input.^9^

The plasma glucose and insulin simulations following LF-UHT and yoghurt ingestion are in good agreement with the measured data using the E-DES-PROT model. In particular, the original E-DES model was less able to capture the measured postprandial glucose data compared to the E-DES-PROT model (Figure 4, AIC, 16.01 and −5.44; BIC, 17.21 and −3.32 for E-DES and E-DES-PROT, respectively, across all challenges). Whereas the first glucose data point after intake (t = 15 min) is accurately captured with the original E-DES model, the remainder of the response is not, and appears to overshoot the measured concentration. The postprandial insulin data were captured well using both models. Looking at the individual level, the E-DES-PROT model was able to capture a wide variety of measured postprandial glucose and insulin responses. Here, the E-DES-PROT model was better able to capture the measured data, for instance for subject 3, 10 (Figure 4A) and subject 3, 5 (Figure 4B). The E-DES-PROT model thus allows capture of more complex meals containing protein as well as carbohydrates, which the original E-DES model was unable to do. The complete overview of the AIC and BIC for the dairy products using the E-DES and E-DES-PROT model is shown in Table S2.

**Figure 5:**
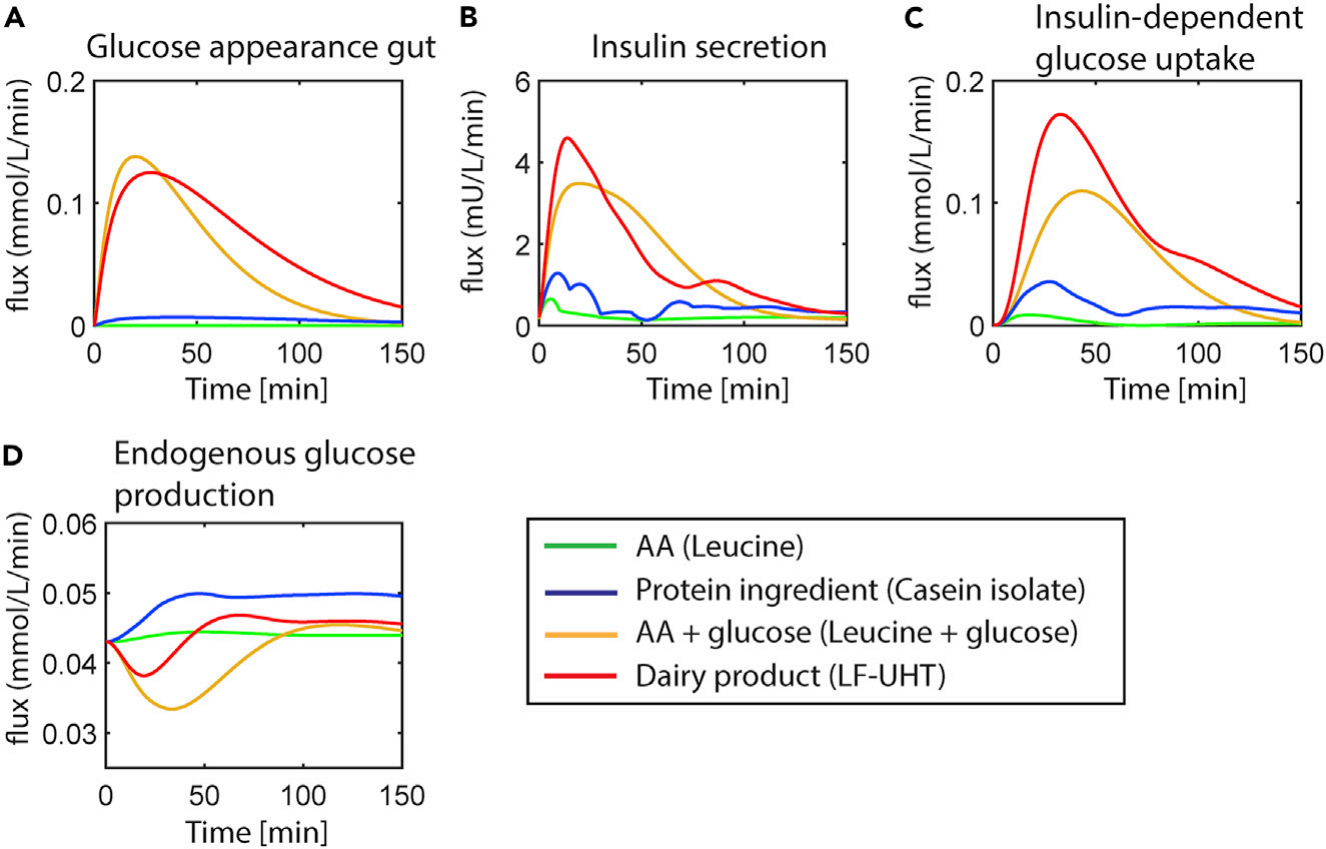
Model fluxes following intake of various meal challenges in the average healthy study populations using the E-DES-PROT model The corresponding model fluxes pertaining to the E-DES-PROT model simulations for leucine (green), micellar casein isolate (blue), leucine + glucose (brown), and LF-UHT (red) intake are shown in panels (A–D).

Model fluxes were compared between E-DES-PROT and the original E-DES model following LF-UHT intake in the average healthy population (Figure S1). The fluxes for endogenous glucose production and insulin-dependent glucose uptake increased more in the E-DES-PROT model compared to the original E-DES model. Despite the small increase in the insulin-dependent glucose uptake flux, a minor change greatly affects the postprandial glucose and insulin concentrations (Figure S2). In addition, model fluxes were compared for different types of meals, ranging from simple AA challenges to more complex dairy products in the average healthy study populations, using the E-DES-PROT model (Figure 5).

The glucose appearance in the gut appears to be more spread out following LF-UHT intake, as compared to leucine + glucose co-ingestion, which has an earlier peak. Insulin secretion and insulin-dependent glucose uptake are substantially lower for leucine and micellar casein isolate ingestion compared to co-ingestion of leucine with glucose and LF-UHT intake, with the largest peak in insulin secretion in the latter. Furthermore, a clear increase from baseline in endogenous glucose production is observed for micellar casein isolate intake, in contrast to LF-UHT and in particular leucine + glucose, which shows the largest decrease from baseline. Leucine ingestion alone only slightly increased endogenous glucose production.

## Discussion

Dietary protein and AAs play an important role in glucose metabolism through stimulating both insulin and glucagon secretion.^13^^,^^15^^,^^16^ In this study, we developed a novel computational model of the glucose-insulin regulatory system, taking the effects of AAs into account, and used this novel model to describe postprandial glucose and insulin dynamics following a variety of simple to complex meals containing AAs and protein. Here, we show that our E-DES-PROT model accurately describes the measured glucose and insulin concentrations, allows insight into the underlying model fluxes, and outperforms the original E-DES model that only takes the postprandial effects of glucose ingestion into account.

The E-DES model by Maas et al.^7^ was selected as a basis for model extension due to its relatively simple description of glucose metabolism. Other models that have previously been reported such as the model of Dalla Man et al.^6^ require data derived from complex, costly measurements (i.e. stable isotope studies) to allow estimation of its model parameters, making it challenging for models to parameterize. In contrast, the E-DES model is less complex in terms of the number of parameters included in the model, and has so far been shown to describe glucose homeostasis in different populations as well as individuals, while including the most important metabolic fluxes.^7^^,^^9^^,^^11^ The present E-DES-PROT model introduces several novel terms accounting for the postprandial effects of both individual and total AAs on glucose and insulin regulation. More specifically, the equation regulating liver glucose production was extended to increase glucose output with increasing plasma AA levels, representing the physiological effects of AAs on glucagon secretion, and consequently hepatic glucose output.^17^ Secondly, the equation regulating pancreatic insulin secretion was extended to increase insulin secretion with increasing plasma AA levels, representing the physiological effects of AAs on β-cells, causing a rise in the ATP/ADP ratio, ultimately leading to the stimulation of insulin granule exocytosis.^18^ These extensions were necessary to capture the characteristics of the postprandial data, while adhering to established human physiology.^17^^,^^19^ To prevent the development of an overly complex model, we modeled these processes using simple linear and derivative terms; in this way, the model can still be readily individualized using standard plasma glucose, insulin, and AA measurements. With the addition of only three parameters, the E-DES-PROT model was able to accurately capture postprandial glucose and insulin data following various challenge tests containing AAs and protein ingredients. The E-DES-PROT model outperforms the original E-DES model in capturing postprandial glucose data, particularly in the case of the dairy challenges, where both AIC and BIC showed a preference for the E-DES-PROT model. For the AA + glucose challenges, both E-DES and E-DES-PROT were able to accurately capture the insulin response, explaining why the AIC and BIC preferred the E-DES model. However, in contrast to the insulin response, the E-DES model was not able to accurately capture the glucose responses. These results confirm the necessity of including the effects of AAs and protein in the models to be able to capture glycemic responses to foods such as to yoghurt. A model based on E-DES that incorporates dietary fat has been developed in parallel and was recently published.^20^ A next step would be to merge these two models into a model able to fully capture the effects of a complex meal, taking into account all three major macronutrient classes (i.e. carbohydrate, protein, and fat).^21^

Despite only slightly improving in capturing the glucose response following AA + glucose challenges, the E-DES-PROT model is physiologically more accurate and provides more detailed insight into the underlying physiological processes (i.e. insulin secretion and endogenous glucose production). Besides being able to describe average postprandial responses to the various challenges, the E-DES-PROT showed the ability to reproduce a wide variety of individual postprandial glucose and insulin responses as well. However, there were some exceptions in which the model did not perfectly capture certain individual postprandial responses. This was observed for responses in which the data points following meal ingestion (t = 0) were below basal glucose concentration (e.g. participant 5, Figure 4B). Furthermore, the model struggled accurately predicting an intermediate dip in the glucose response (e.g. participant 5, Figure 4A).

The mechanistic nature of the model also allows the investigation of non-measured variables such as metabolite fluxes between tissues. Inspecting the metabolite fluxes, we found that there was an increase in insulin-dependent glucose uptake using the E-DES-PROT model compared to the original E-DES model, resulting in accurate description of the postprandial glucose data. The model fluxes calculated for various meals included in this study provide information on physiological processes underlying the dynamic responses. For example, glucose appearance in the gut seems to be more spread out for the dairy product (i.e. LF-UHT) compared to the simpler AA + glucose co-ingestion test (i.e. leucine + glucose). Furthermore, endogenous glucose production was increased for protein-only meals (i.e. micellar casein isolate), corresponding with findings from literature.^15^^,^^22^ While beyond the scope of the present study, investigating model parameters and corresponding fluxes at the individual level with the new E-DES-PROT model might provide further insight into the glucometabolic status of individuals.

In conclusion, we present a new physiology-based computational model of the glucose homeostasis that extends the E-DES model with the postprandial effects of AAs and protein. The E-DES-PROT model allows, for the first time, to accurately describe postprandial responses following different AA challenges (with and without co-ingestion of glucose), dried milk protein ingredients, and dairy challenges, and is able to provide information on physiological processes underlying the meal responses. Introducing AAs in these models is important to move toward describing physiologically relevant complex meals. In addition, our model outperforms the original E-DES model in terms of describing postprandial glucose responses following dairy products. As the model covers two out of three macronutrient classes (carbohydrates and protein), future studies should explore the possibility to further extend the E-DES-PROT model with fat to allow model-based prediction of glucose responses to complex meals varying in macronutrient composition and content.

### Limitations of the study

The increased liver glucose output was modeled to be dependent on the AA concentration in the plasma. However, AAs are known to stimulate glucagon secretion, which in turn increases liver glucose output.^17^ As glucagon is not explicitly accounted for in the E-DES model, future work should consider incorporating glucagon in the E-DES model, as has been implemented before in the Dalla Man model.^23^ Secondly, as the objective of our research was to quantify the effect of AAs on postprandial glucose-insulin dynamics, a forcing function is used to describe the rate of appearance of AAs in E-DES-PROT. In future research, the addition of a function to explicitly describe the rate of appearance could increase the functionality of our model. This rate of appearance function would allow simulation of plasma AAs, without the need for measured plasma AAs to be provided as input. Furthermore, this would allow refinement of the glucose rate of appearance, as protein (and fat) has been known to delay gastric emptying.^24^

Individual AAs have been shown to have distinct effects on the glucose and insulin response,^13^^,^^19^ but also interact with each other when provided together.^25^ In this study, we added up the AA profiles (tAA) for the protein ingredients and dairy products, and did not include possible interactions between individual AAs in the E-DES-PROT model. Furthermore, not only AAs but also fat influences the blood glucose response in response to complex meals.^26^^,^^27^ However, incorporating the postprandial effects of fat on glucose metabolism was beyond the scope of this present study. Identifiability analysis showed that the parameters related to AAs (*k*11–*k*13) were identifiable for AA challenges and milk protein ingredients (examples are shown in Figure S3). However, for the AA + glucose challenges as well as for the dairy products, only the parameters *k*1, *k*5, *k*6, *k*8, and *k*13 were consistently deemed identifiable. The unidentifiability of the *k*11 and *k*12 parameter in several of these challenges might have resulted from functional relationships between parameters.

## STAR★Methods

### Key resources table

**Table:** 

REAGENT or RESOURCE	SOURCE	IDENTIFIER
**Software and algorithms**		

MATLAB 2018b	The MathWorks Inc.	https://nl.mathworks.com/products/matlab.html

**Other**		

Original E-DES-PROT Model code	This paper	Data S1, https://github.com/BartvSloun/E-DES-PROT

### Resource availability

#### Lead contact

Further information and requests for resources should be directed to and will be fulfilled by the lead contact Bart van Sloun (bart_van_sloun@hotmail.com).

#### Material availability

No new materials or reagents were generated during this study.

### Method details

#### Study workflow

The study workflow is illustrated in Figure S4. Briefly, the existing E-DES model was extended to a model that accounts for the postprandial effects of AAs and protein on glucose and insulin dynamics. Model equations were adjusted and additional parameters were introduced to take the effects of AAs on insulin secretion and liver glucose production, as observed from literature, into account. Subsequently, postprandial time-series data, extracted from the literature,^13^ and obtained from a previously performed randomized, single-blind crossover trial in healthy elderly males and females (RCT; NCT02546141)^14^ were used to estimate the model parameters. The ability of the model to describe the measured data was evaluated using the sum of squared residuals (SSR), the Akaike Information Criterion (AIC) and the Bayesian Information Criterion (BIC). Model fluxes were compared between the E-DES and the newly developed E-DES-PROT model, as well as for various meal challenges.

#### Collection of data

Publicly available datasets, containing postprandial time-series data of AAs, glucose, and insulin following various AA challenge tests (leucine, isoleucine, lysine, glycine, proline, and phenylalanine; with or without glucose) in healthy individuals were included in the present study (summarized in^13^). In all experiments, plasma samples were taken from the antecubital vein in the fasting state (t=0) and 10, 20, 30, 40, 50, 60, 70, 80, 90, 100, 110, 120, and 150 minutes after ingestion of 1 mmol AA per kg of lean body weight (with or without 25g glucose). In addition, we used data on postprandial AAs (arginine, glutamine, serine, asparagine, glycine, threonine, alanine, methionine, proline, lysine, aspartic acid, histidine, valine, glutamic acid, tryptophan, leucine, phenylalanine, isoleucine, cysteine and tyrosine), glucose, and insulin time-series from a randomized single-blind crossover trial (RCT; NCT02546141), in which ten participants (five male) received two spray dried milk protein ingredients (whey protein concentrate, WPC; micellar casein isolate, MCI) and six dairy products (low-fat untreated milk (LF-UHT); low-fat pasteurized milk (LF-PAS); full-fat untreated milk (FF-UHT); full-fat pasteurized milk (FF-PAS); low-fat yoghurt; full-fat cheese) in random order, as previously described.^14^ The dairy products and protein ingredients were supplied on eight separate test days, with a one-week washout period in between. For each meal, an appropriate amount of the product to ensure 25g of protein intake was consumed. For the milk protein ingredients (i.e. WPC and MCI), this was achieved by dissolving an appropriate amount of powder in water to attain a solution of 700mL containing 25g of protein. To standardize the volume for all products, water was added to a total of 700mL of volume ingested. Plasma samples were taken from the antecubital vein in the fasting state (t=0) and 15, 30, 45, 60, 75, 90, 105, 120, 150, 180, 210, 240, and 300 minutes after ingesting the protein ingredients and dairy products. An overview of the datasets included in the present study is given in Table S3.

#### Development of a novel physiology-based computational model of glucose homeostasis

The Eindhoven Diabetes Education Simulator (E-DES, version 1.1) published by Maas et al.^7^^,^^9^ formed the basis for the model extension with AAs in the present study. The E-DES model is a physiology-based computational model of the glucose regulatory system in healthy individuals and patients with type 1 and type 2 diabetes.^10^ It consists of a system of coupled differential equations, which describe the change of the mass or concentration of either glucose or insulin over time. Each of these equations consists of a positive inflow and negative outflow term and can be summarized as follows: (i) glucose balance in the gut is determined through the inflow of glucose mass from the stomach and glucose leaving the gut through uptake by the plasma (ii) glucose balance in the plasma is determined by glucose inflow from the gut in conjunction with glucose output from the liver and glucose uptake by insulin-(in)dependent tissues (iii) insulin balance in the plasma is determined by inflow of endogenously produced insulin from the pancreas and uptake of insulin by the interstitial fluid (iv) insulin balance in the interstitial fluid is determined by insulin inflow from the plasma and removal of insulin from the interstitial fluid proportional to the interstitial insulin fluid concentration. The rates through which these processes occur are controlled by parameters (denoted with *k*), which have been estimated and validated on multiple oral glucose tolerance tests (OGTTs) in healthy populations.^10^ The model parameters are described in Table S4. The model inputs, equations, fluxes, constants are described in detail in Data S4.

#### Model development

In this study, we extended the previously developed E-DES model to also account for the postprandial effects of AAs on glucose and insulin dynamics (illustrated in Figure S5). Firstly, the equation regulating glucose production from the liver (Equation 1) was extended with a proportional (*k*11) term to accommodate an increase in liver glucose production proportional to the AA concentration present in the plasma (AA^pl^(t)) relative to the basal concentration (AA_b_^pl^).

(Equation 1) $$g^{liv}\left(t\right)=g_{b}^{liv}-k_{3}\left(G^{pl}\left(t\right)-G_{b}^{pl}\right)-k_{4}\beta \left(I^{if}\left(t\right)\right)+k_{11}\left({AA}^{pl}\left(t\right)-{AA}_{b}^{pl}\right)$$

Secondly, the equation regulating insulin secretion from the pancreas (Equation 2) was extended with a derivative (*k*12) and proportional (*k*13) term to accommodate an increase in insulin secretion (i) based on the rate of change of plasma AAs ($\frac{dAA^{pl}}{dt})$, and (ii) proportional to the AA concentration present in the plasma (AA^pl^(t)) relative to the basal concentration (AA_b_^pl^).

(Equation 2) $$i^{pnc}(t)=\beta^{-1}({k_{6}(G}^{pl}{\left(t\right)-G}_{b}^{pl})+\left(\frac{k_{7}}{\tau_{i}}\right)\int {(G}^{pl}(t)-G_{b}^{pl})dt+\left(\frac{k_{7}}{\tau_{i}}\right)G_{b}^{pl}+(k_{8}\tau_{d})\frac{dG^{pl}}{dt}+k_{12}\frac{d{AA}^{pl}}{dt}+k_{13}({AA}^{pl}(t)-{AA}_{b}^{pl}))$$

The extended Equations 1 and 2 described above require plasma AA concentrations as model input. Therefore, measured AA concentrations following the challenge tests were interpolated via a fitted piecewise cubic Hermite interpolating polynomial (pchip), and provided to the model as AA^pl^(t). For the RCT (NCT02546141), the following AA measurements were added up, interpolated, and denoted as total AA (tAA): arginine, glutamine, serine, asparagine, glycine, threonine, alanine, methionine, proline, lysine, aspartic acid, histidine, valine, glutamic acid, tryptophan, leucine, phenylalanine, isoleucine, cysteine, and tyrosine.

#### Model calibration

Model calibration was performed by generating parameter values that resulted in an optimal description of measured data. This was done through minimizing a cost function, representing the sum of squared residual (SSR) in the model prediction for glucose and insulin (Equation 3). The SSR is minimized using *lsqnonlin*, a local, gradient-based least squares solver in MATLAB (Version R2018b). Optimal parameter sets were obtained using twenty-five initializations of the optimization algorithm with 25% random noise starting from the original parameter value for the average healthy population.^9^

(Equation 3) $$SSR=\sum_{j=1}^{m}\sum_{i=1}^{N}{\left(\gamma \left(\left(y_{i,j}|\vec{\theta }\right)-d_{i,j}\right)\right)}^{2}$$

Where *m*, and *N* represent the number of metabolites and the number of time-points, respectively. The measured data is denoted by *d*, while *y* is the corresponding model prediction given the parameter vector $\vec{\theta }$. A weight factor *γ =* 0.1 was used in the case of insulin (*γ* = 1 in case of glucose) to account for the unit difference (mmol/L, mU/L for glucose and insulin, respectively) between the molecules. As the *lsqnonlin* function, that minimizes the sum of squared error, does this simultaneously for glucose and insulin, the *γ* factor aims to bring the units for glucose and insulin closer together to avoid prioritizing one or the other in the optimization process.

#### Model selection and analysis

Visual inspection was performed to evaluate the goodness-of-fit of the simulated glucose and insulin responses to the measured data. In order to compare the E-DES and the E-DES-PROT model, we selected the parameters identified from our previous work.^11^ In that work, a systematic model selection pipeline was implemented to allow personalization of the E-DES model through reducing the number of parameters to be estimated, resulting in a model containing parameters *k*1, *k*5, *k*6, and *k*8 (sensitivity is shown in Figure S6). In the current work, we estimated those parameters, both for the systematic review datasets and the randomized single-blind crossover trial. The selected parameters represent distinct physiological processes involved in glucose and insulin regulation, described in Table S4. For the E-DES-PROT model simulation, the AA parameters (*k*11*-k*13) were also estimated. Parameters G_b_^pl^ and I_b_^pl^ (sensitivity is shown in Figure S7) were set to be equal to the first data-point (t = 0 min) of the measured responses, whereas the other parameters were set to the average healthy population values from the original publication.^9^

Model performance was evaluated using the Akaike Information Criterion (AIC) and the Bayesian Information Criterion (BIC), in which model complexity (i.e. number of estimated parameters) was penalized (Equations 4 and 5 respectively).

(Equation 4) $$AIC=N*\ln\left(\frac{SSR}{N}\right)+2*K$$

(Equation 5) $$BIC=N*\ln\left(\frac{SSR}{N}\right)+\ln\left(N\right)*\;K$$

N represents the number of observations, and K the number of parameters. Given a set of candidate models that describe the postprandial time-series data, the preferred model is the one with the lowest AIC and BIC value, indicating the better-fit model whilst taking the number of parameters into account. In addition, model fluxes were calculated and compared between the E-DES and E-DES-PROT model, as well as for the various meal challenges. Parameter identifiability was assessed using Profile Likelihood Analysis (PLA). In PLA, the value of one parameter is changed iteratively from its optimal value and the remaining parameters are re-estimated. An increase in the cost function for the model fit indicates that a reliable parameter estimate has been obtained and the parameter is identifiable given the model structure and data.

#### Computer software

The model was implemented and analyzed in MATLAB (MATLAB, Version R2018b, The Mathworks, Inc., Natick, Massachusetts, United States). The ordinary differential equation model was simulated using the variable step solver ode15s.

## Data Availability

• The data of the randomized single-blind crossover trial study (NCT02546141) are available to eligible researchers from Thom Huppertz (Thom.Huppertz@frieslandcampina.com). • All original code is provided in the supplementary materials (Data S1) and has also been deposited in GitHub (https://github.com/BartvSloun/E-DES-PROT). • All additional information required to re-analyze the data reported in this paper is available from the lead contact upon request

## Supporting citations

The following references are included in the supplemental information (i.e. Table S3): 28, 29, 30, 31, 32, 33.

## Supplemental information

Document S1. Figures S1–S7, Data S2–S4, and Tables S1–S8

Data S1. MATLAB implementation of the E-DES-PROT model used in the manuscript, related to STAR Methods
