# Supplementary material for: E-DES-PROT: A novel computational model to describe the effects of amino acids and protein on postprandial glucose and insulin dynamics in humans
Source: iScience. 2023 Feb 18;26(3):106218. doi: 10.1016/j.isci.2023.106218 (PMC9989689; doi:10.1016/j.isci.2023.106218)
Supplement: Document S1. Figures S1–S7, Data S2–S4, and Tables S1–S8 [file mmc1.pdf]

iScience, Volume *26*

Supplemental information

E-DES-PROT: A novel computational model

to describe the effects of amino acids and protein

on postprandial glucose and insulin dynamics in humans

Bart van Sloun, Gijs H. Goossens, Balázs Erdõs, Shauna D. O'Donovan, Cécile M. Singh-Povel, Jan M.W. Geurts, Natal A.W. van Riel, and Ilja C.W. Arts

Supplemental Table S1: Sum of squared residuals (SSR), Akaike Information Criterion (AIC), and Bayesian Information Criterion (BIC) of the AA+glucose challenges from literature datasets using the E-DES and E-DES-PROT model, related to STAR Methods.

**E-DES**	**SSR**	**AIC**	**BIC**	**E-DES-PROT**	**SSR**	**AIC**	**BIC**
**Glucose + Insulin response**				**Glucose + Insulin response**			
Leucine + Glucose	0.9853	-77.0958	-72.0634	Leucine + Glucose	0.8266	-75.6618	-66.8551
Isoleucine + Glucose	4.2394	-39.1555	-34.1231	Isoleucine + Glucose	4.7099	-30.4194	-21.6125
Lysine + Glucose	2.1334	-57.0094	-51.9771	Lysine + Glucose	1.5095	-60.0042	-51.1976
Phenylalanine + Glucose	3.3157	-45.5451	-40.5127	Phenylalanine + Glucose	3.4446	-38.5533	-29.7468
Glycine + Glucose	1.7085	-59.0821	-54.0498	Glycine + Glucose	2.0023	-45.6105	-37.3638
Proline + Glucose	1.6715	-63.3544	-58.3221	Proline + Glucose	1.5434	-59.4260	-50.6202
Across all co-ingestion tests	14.0537	-7.9954	-2.9630	Across all co-ingestion tests	14.0364	-2.0275	6.7792
**E-DES**	**SSR**	**AIC**	**BIC**	**E-DES-PROT**	**SSR**	**AIC**	**BIC**
**Glucose response**				**Glucose response**			
Leucine + Glucose	0.5345	-33.4877	-31.2279	Leucine + Glucose	0.3426	-33.2711	-29.3164
Isoleucine + Glucose	3.1633	-10.3733	-8.1135	Isoleucine + Glucose	0.6546	-24.8533	-20.8982
Lysine + Glucose	0.4056	-37.0743	-34.8145	Lysine + Glucose	0.5807	-26.4106	-22.4555
Phenylalanine + Glucose	2.4123	-13.8971	-11.6373	Phenylalanine + Glucose	1.6784	-12.6121	-8.6578
Glycine + Glucose	0.9126	-22.9169	-20.6571	Glycine + Glucose	0.5694	-22.5770	-19.1826
Proline + Glucose	0.1894	-46.9771	-44.7173	Proline + Glucose	0.1350	-45.3792	-41.4219

Across all co-ingestion tests	7.6176	1.0517	3.3115	Across all co-ingestion tests	3.9607	-1.4509	2.5038
**E-DES**	**SSR**	**AIC**	**BIC**	**E-DES-PROT**	**SSR**	**AIC**	**BIC**
**Insulin response**				**Insulin response**			
Leucine + Glucose	0.4508	-35.7023	-33.4425	Leucine + Glucose	0.4840	-28.7772	-24.8226
Isoleucine + Glucose	1.0761	-24.3910	-22.1312	Isoleucine + Glucose	4.0553	-1.1440	2.8106
Lysine + Glucose	1.7278	-18.2353	-15.9755	Lysine + Glucose	0.9288	-20.3042	-16.3499
Phenylalanine + Glucose	0.9034	-26.6643	-24.4045	Phenylalanine + Glucose	1.7662	-11.9496	-7.9949
Glycine + Glucose	0.7959	-24.5583	-22.2985	Glycine + Glucose	1.4329	-11.5027	-8.1081
Proline + Glucose	1.4821	-20.2295	-17.9697	Proline + Glucose	1.4085	-14.8917	-10.9369
Across all co-ingestion tests	6.4361	-1.1393	1.1205	Across all co-ingestion tests	10.0757	10.6873	14.6419

Supplemental Table S2: Sum of squared residuals (SSR), Akaike Information Criterion (AIC), and Bayesian Information Criterion (BIC) of the dairy challenges of the randomized single-blind crossover trial study dataset using E-DES and E-DES-PROT model, related to STAR Methods.

**E-DES**	**SSR**	**AIC**	**BIC**	**E-DES-PROT**	**SSR**	**AIC**	**BIC**
**Glucose + Insulin response**				**Glucose + Insulin response**			
LF-UHT	4.7395	-16.0199	-12.4584	LF-UHT	0.5840	-47.7081	-41.4755
LF-PAS	3.7143	-20.4073	-16.8458	LF-PAS	0.2709	-61.5370	-55.3022
FF-UHT	6.1975	-11.1921	-7.6306	FF-UHT	0.7732	-42.6566	-36.4240
FF-PAS	5.7793	-12.4495	-8.8881	FF-PAS	0.8822	-40.2827	-34.0501
Yoghurt	5.3701	-13.7713	-10.2100	Yoghurt	1.3470	-32.6649	-26.4323
Across all challenges	25.8008	14.4806	18.0421	Across all challenges	3.8573	-13.7273	-7.4947
**E-DES**	**SSR**	**AIC**	**BIC**	**E-DES-PROT**	**SSR**	**AIC**	**BIC**
**Glucose response**				**Glucose response**			
LF-UHT	4.4449	-0.1083	1.1021	LF-UHT	0.2395	-23.3196	-21.1998
LF-PAS	3.2612	-3.2048	-1.9946	LF-PAS	0.1317	-29.2981	-27.1800
FF-UHT	5.3515	1.7479	2.9583	FF-UHT	0.3138	-20.6166	-18.4977
FF-PAS	5.0361	1.1406	2.3508	FF-PAS	0.4418	-17.1954	-15.0767
Yoghurt	4.1821	-0.7177	-0.4926	Yoghurt	0.3043	-20.9242	-18.8052
Across all challenges	22.2758	16.0092	17.2195	Across all challenges	1.4311	-5.4414	-3.3233

E-DES	SSR	AIC	BIC	E-DES-PROT	SSR	AIC	BIC
**Insulin response**				**Insulin response**			
LF-UHT	0.2946	-18.4126	-18.0949	LF-UHT	0.3446	-11.1585	-10.6024
LF-PAS	0.4530	-14.9704	-14.6527	LF-PAS	0.1392	-18.4103	-17.8542
FF-UHT	0.8460	-16.6983	-15.4879	FF-UHT	0.4595	-8.8565	-8.3004
FF-PAS	0.7432	-11.0099	-10.6921	FF-PAS	0.4404	-9.1956	-8.6400
Yoghurt	1.1881	-7.2567	-6.9389	Yoghurt	1.0427	-2.3009	-1.7449
Across all challenges	3.5249	1.4433	1.7611	Across all challenges	2.4264	4.4557	5.0118

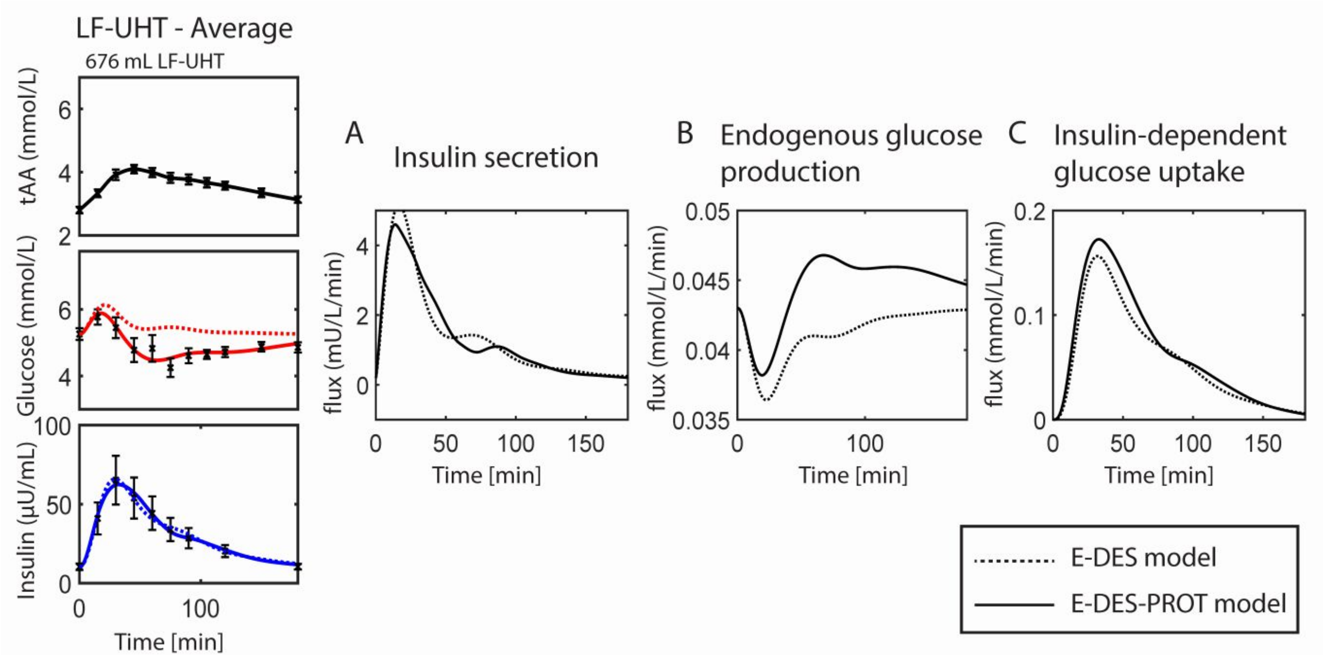

Supplemental Figure S1: Simulated model fluxes following LF-UHT intake, related to Figure 4. The leftmost column pertains to the average LF-UHT simulation. Panel A, B, C, describe the insulin secretion, endogenous glucose production and insulin-dependent glucose uptake flux, respectively, using the original E-DES model (dashed black) and the E-DES-PROT model (black)

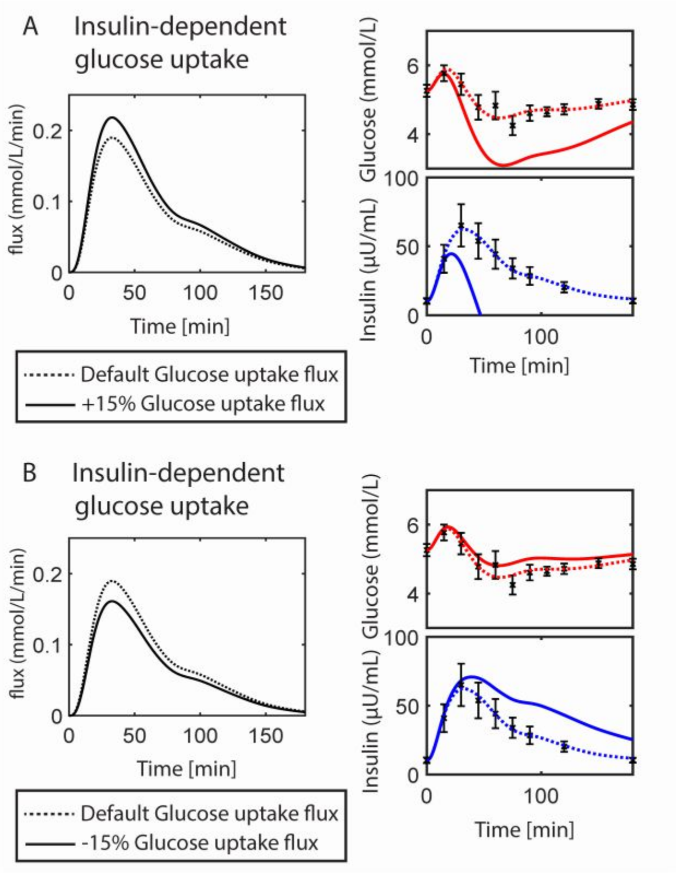

Supplemental Figure S2: Varying the insulin-dependent glucose uptake flux following LF-UHT intake, related to Figure 4. Panel A and B show a 15% increase and decrease in the insulin-dependent glucose uptake flux, with the corresponding model simulation in red and blue for glucose and insulin, respectively.

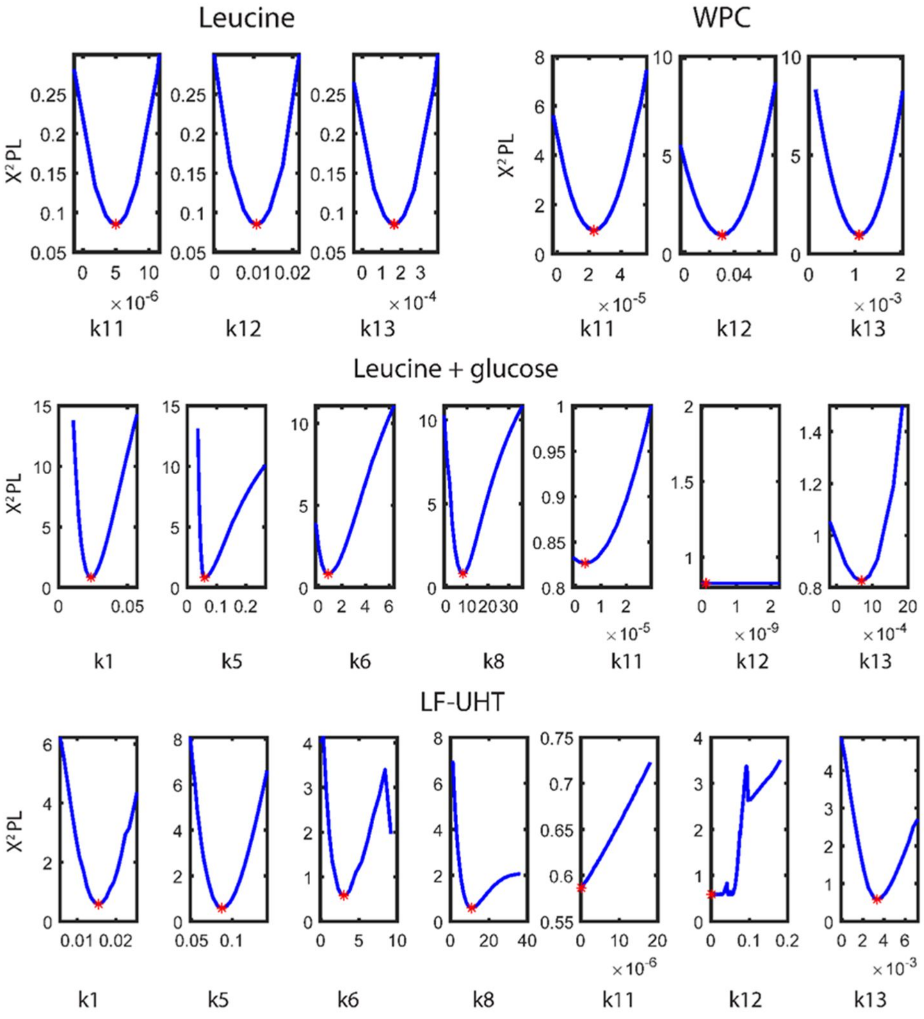

Supplemental Figure S3: Profile likelihood analysis of leucine, WPC, leucine+glucose, and LF-UHT intake, related to STAR Methods. The red asterisk indicates the SSR of the model fitted using the optimal parameter values estimated from data, while the blue line corresponds to the error as the other parameter values are being re-estimated after adjusting the parameter value iteratively.

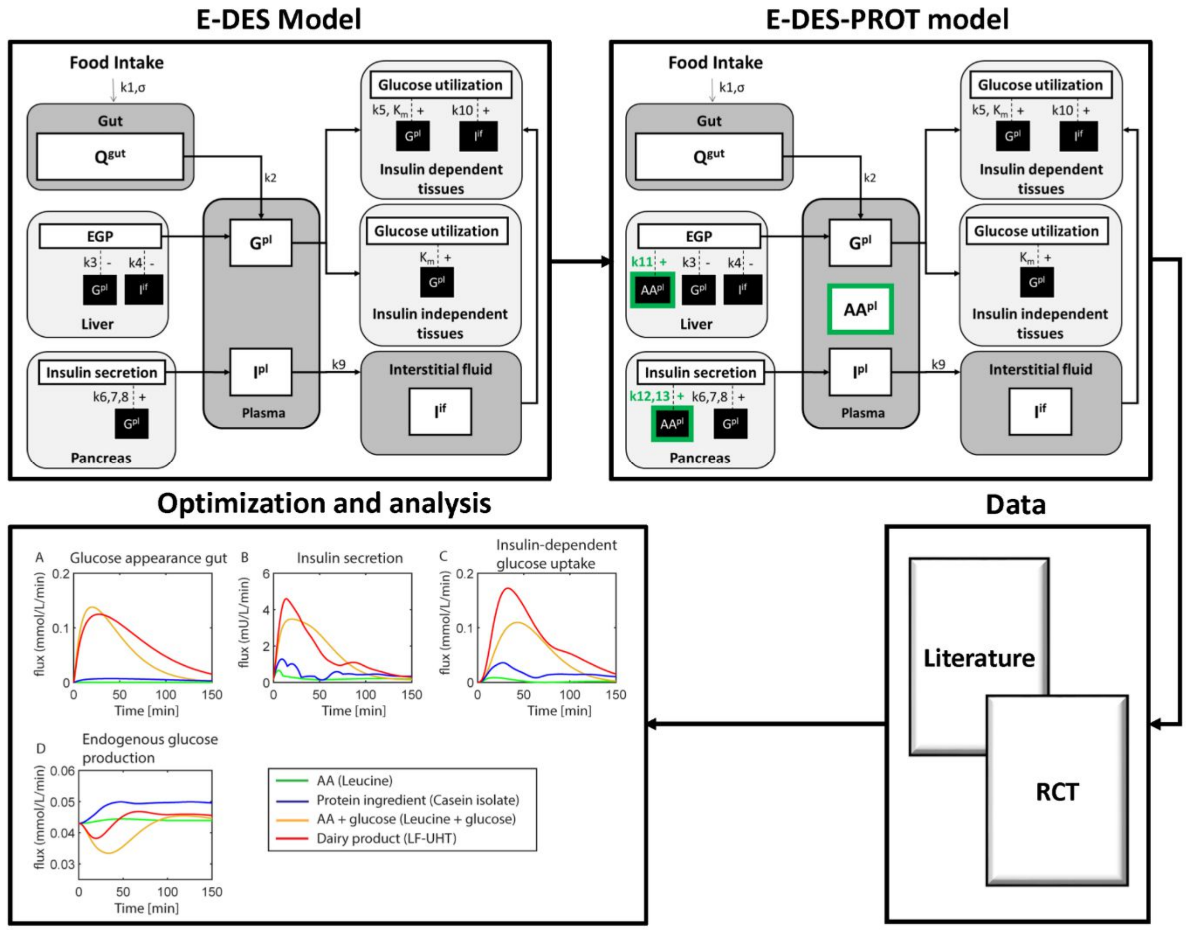

Supplementary Figure S4: Overview of the study workflow, related to STAR Methods. The E-DES model was extended (E-DES-PROT) to account for the postprandial effects of AAs and protein on glucose and insulin dynamics. Model equations were adjusted and additional parameters were introduced. Postprandial AA, glucose, and insulin time-series data from the literature and the randomized, single-blind crossover trial (RCT; NCT02546141) were used for parameter estimation. The models were evaluated using the sum of squared residuals (SSR), the Akaike Information Criterion (AIC) and Bayesian Information Criterion (BIC). Model fluxes were compared between the E-DES and the newly developed E-DES-PROT model, as well as for various meal challenges.

Supplementary Table S3: Overview of the datasets, related to STAR Methods.

Author	Challenge	Dosage	N	M/F	Mean Age (yr)	Mean BMI (kg/m^2)	#G	#I	#AA
Kalogeropoulou (Kalogeropoulou et al., 2008)	AA	7g Leucine	13	6/7	24	24	14	14	5
	AA+Glucose	7g Leucine + 25g Glucose	13	6/7	24	24	14	14	5
Nuttall (Nuttall et al., 2008)	AA	7.4g Isoleucine	9	3/6	33.8	28	14	14	5
	AA+Glucose	7.4g Isoleucine + 25g Glucose	9	3/6	33.8	28	14	14	5
Kalogeropoulou (Kalogeropoulou et al., 2009)	AA	11g Lysine	13	6/7	30	26	14	14	5
	AA+Glucose	11g Lysine + 25g Glucose	13	6/7	30	26	14	14	5
Gannon (Gannon et al., 2002)	AA	4.6g Glycine	9	5/4	21-52	25.9	13	13	5
	AA+Glucose	4.6g Glycine +25g Glucose	9	5/4	21-52	25.9	13	13	5
Nuttall (Nuttall et al., 2004)	AA	6.0g Proline	8	4/4	28	23	14	14	5
	AA+Glucose	6.0g Proline + 25g Glucose	8	4/4	28	23	14	14	5
Nuttall (Nuttall et al., 2006)	AA	9.7g Phenylalanine	6	3/3	26	24	14	14	5
	AA+Glucose	9.7g Phenylalanine + 25g Glucose	6	3/3	26	24	14	14	5

Horstman (Horstman *et al.*, 2021)	Protein ingredient	31g WPC: 24.8g protein, 2g carbohydrate, 1.6g fat	10	5/5	66.7± 4.3	25.6± 2.6	14	9	14
	Protein ingredient	29g MCI: 25.2g protein, 2.9g carbohydrate, 0.3g fat	10	5/5	66.7± 4.3	25.6± 2.6	14	9	14
	Dairy product	676 mL LF-UHT: 25g protein, 33.8g carbohydrate, 0.7g fat	10	5/5	66.7± 4.3	25.6± 2.6	14	9	14
	Dairy product	694 mL LF-PAS: 25g protein, 32.6g carbohydrate, 0.7g fat	10	5/5	66.7± 4.3	25.6± 2.6	14	9	14
	Dairy product	694 mL FF-UHT: 25g protein, 32.6g carbohydrate, 25g fat	10	5/5	66.7± 4.3	25.6± 2.6	14	9	14
	Dairy product	694 mL FF-PAS: 25g protein, 32.6g carbohydrate, 25g fat	10	5/5	66.7± 4.3	25.6± 2.6	14	9	14
	Dairy product	532 mL Yoghurt: 25g protein, 21.3g carbohydrate, 0g fat	10	5/5	66.7± 4.3	25.6± 2.6	14	9	14
	Dairy product	97g Cheese: 24.9g protein, 0g	10	5/5	66.7± 4.3	25.6± 2.6	14	9	14

	carbohydrate,<br>32.5g fat	

Abbreviations are: BMI: Body Mass Index; #G: Number of glucose measurement timepoints; #I: Number of insulin measurement timepoints; #AA: Number of AA measurement timepoints; N: Number of study participants; M: Males; F: Females; WPC: Whey protein concentrate; MCI: Micellar casein isolate; LF-UHT: Low-fat untreated treated milk; LF-PAS: Low-fat pasteurized milk; FF-UHT: Full-fat untreated-treated milk; FF-PAS: Full-fat pasteurized milk

Supplementary Table S4: Overview of the E-DES-PROT model parameters, related to STAR Methods.

**Name**	**Description**	**Units**
$k1$	Rate constant of glucose appearance in the gut	1/min
$k2$	Rate constant of gut emptying	1/min
$k3$	Rate constant of ΔG suppression of EGP when $G^{pl} > G_b^{pl}$	1/min
$k4$	Rate constant of I^{if}-dependent suppression of EGP	1/min
$k5$	Rate constant of insulin-dependent glucose uptake	1/min
$k6$	Rate constant of ΔG dependent insulin production	1/min
$k7$	Rate constant of ∫G dependent insulin production	1/min
$k8$	Rate constant of $\frac{dG}{dt}$ dependent insulin production	1/min
$k9$	Rate constant of insulin outflow from plasma to interstitial fluid	1/min
$k10$	Rate constant of interstitial fluid insulin utilization	1/min
**$k11$**	**Rate constant of ΔAA increase of EGP when $AA^{pl} > AA_b^{pl}$**	**1/min**
**$k12$**	**Rate constant of $\frac{dAA}{dt}$ dependent insulin production**	**1/min**
**$k13$**	**Rate constant of ΔAA dependent insulin production**	**1/min**
σ	Shape factor of the gastric emptying pattern	dimensionless
K_m	Michaelis-Menten constant for glucose uptake	mg/dL

$k11$-$k13$ are included as additional parameters for the E-DES-PROT model, indicated in bold. Abbreviations are: G: Glucose; pl: Plasma; b: Basal; I: Insulin; if: Interstitial fluid; AA: Amino acids.

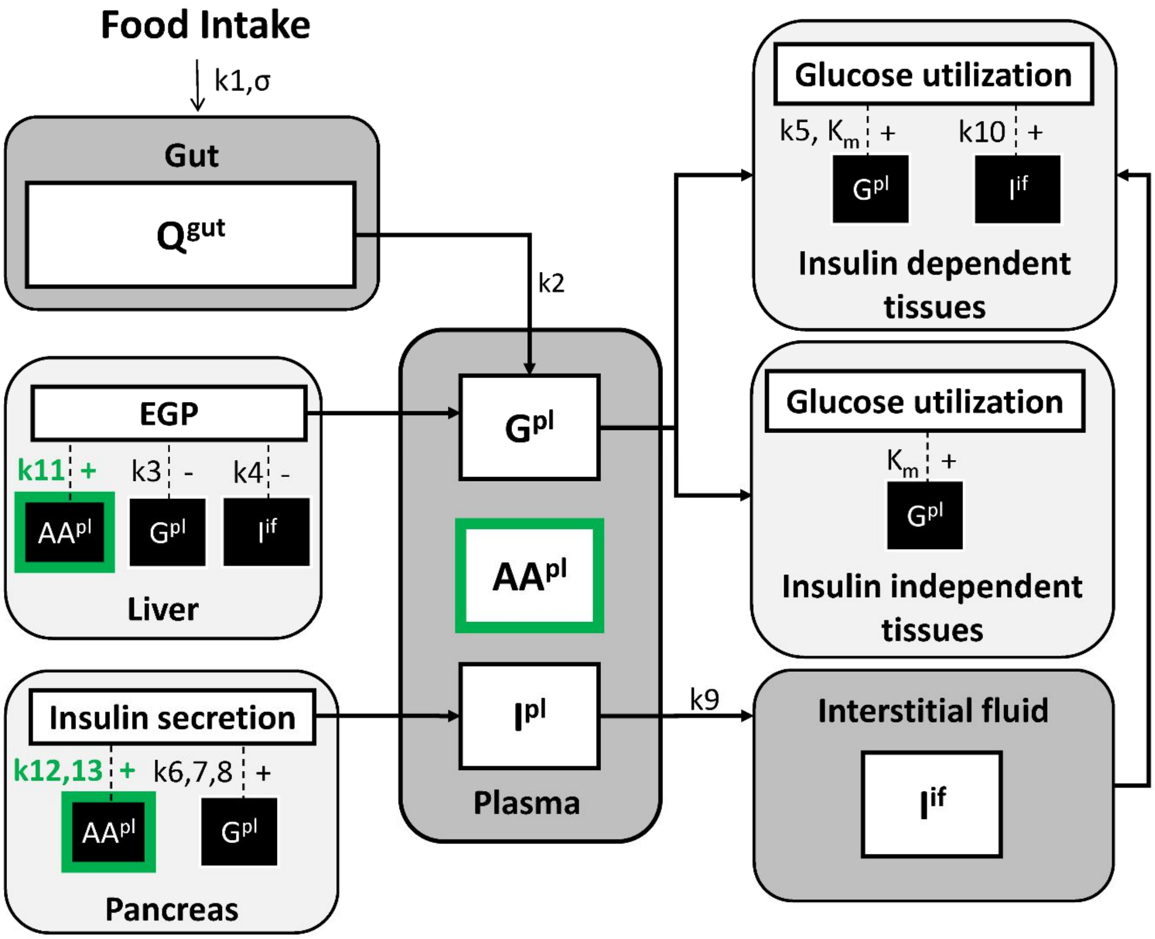

Supplementary Figure S5: Schematic representation of the E-DES-PROT model, related to STAR Methods. The dark-gray areas show the three model compartments (i.e. the gut, plasma, and interstitial fluid) used in the model. The black arrows denote the model fluxes, with corresponding parameters denoted with k. In the model, glucose enters the system via simulated ingestion in the gut (Qgut). Plasma amino acid (AApl) concentration are provided as input via fitted piecewise cubic Hermite interpolating polynomial of the measured AA data. Full model equations are described in Data S4.

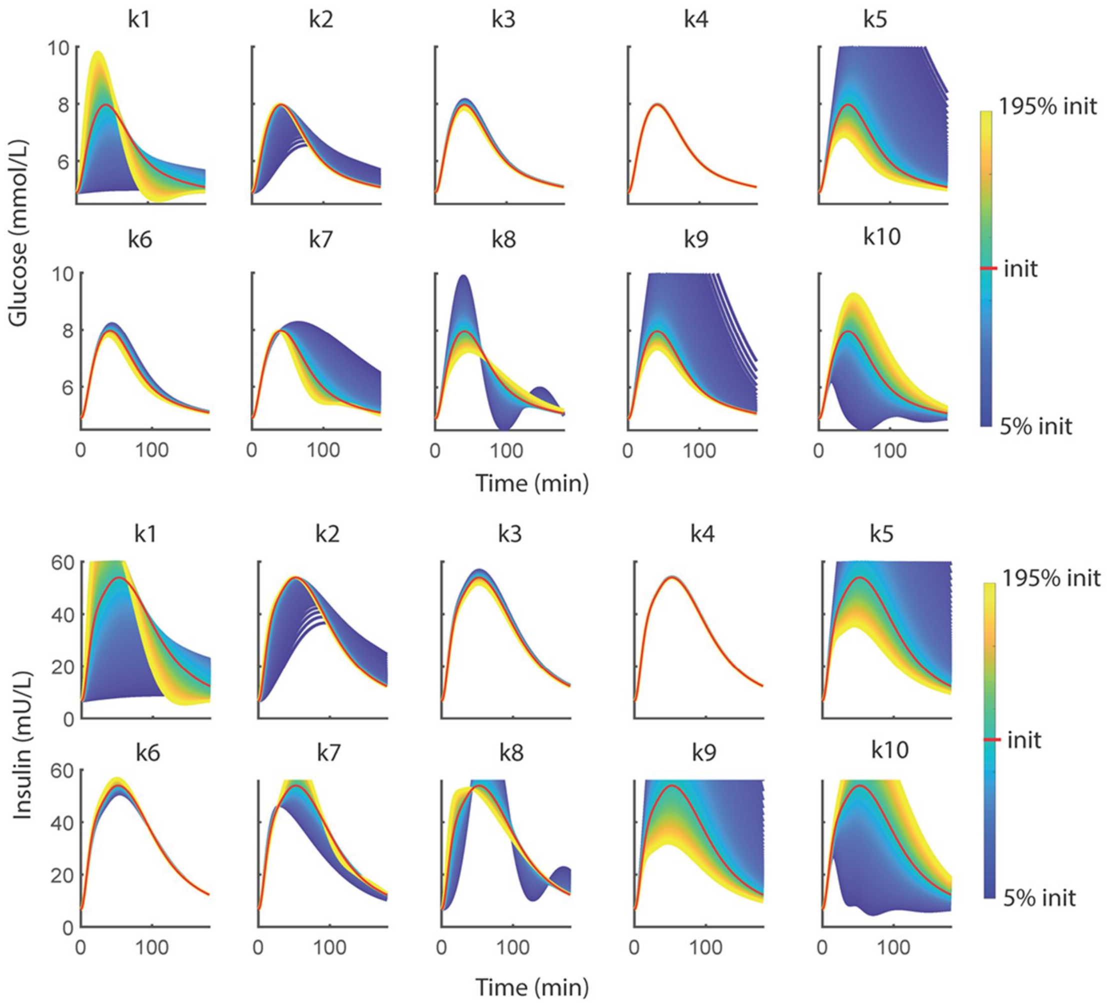

Supplemental Figure S6: Local parameter sensitivity analysis on the simulated plasma glucose and insulin response following 75g glucose ingestion in the E-DES model, related to STAR Methods. The E-DES model parameters are varied in both directions while maintaining others at a constant value. A threshold of 95% in both directions compared to the average healthy population values was selected as the limit of sensitivity.

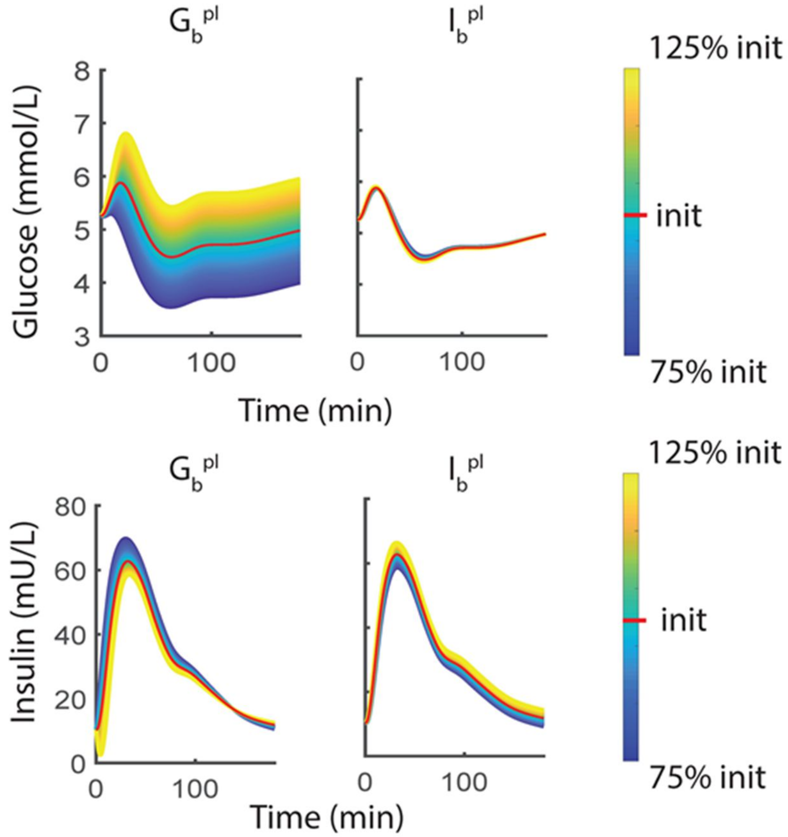

Supplemental Figure S7: Gbpl and Ibpl sensitivity in the E-DES-PROT model, related to STAR Methods. Gbpl and Ibpl were varied in both directions. A threshold of 25% in both directions compared to the average healthy population values was selected as the limit of sensitivity.

Data S2: Simulated postprandial responses for all individuals in the randomized single-blind crossover trial study following ingestion of protein ingredients (WPC, MCI) and cheese, related to Figure 2.

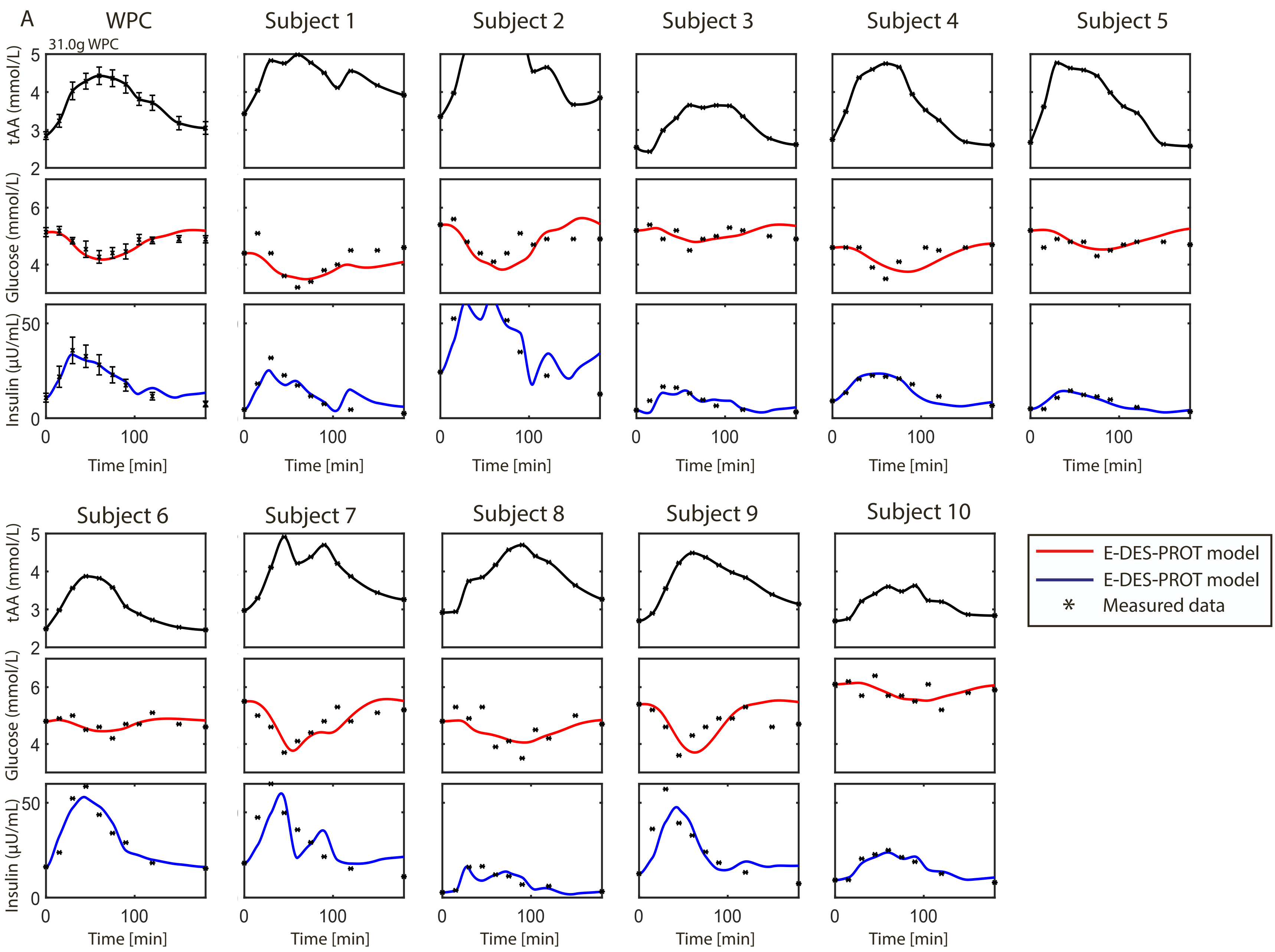
A
WPC
31.0g WPC
Subject 1
Subject 2
Subject 3
Subject 4
Subject 5
Subject 6
Subject 7
Subject 8
Subject 9
Subject 10
tAA (mmol/L)
Glucose (mmol/L)
Insulin (μU/mL)
Time [min]
E-DES-PROT model
E-DES-PROT model
Measured data

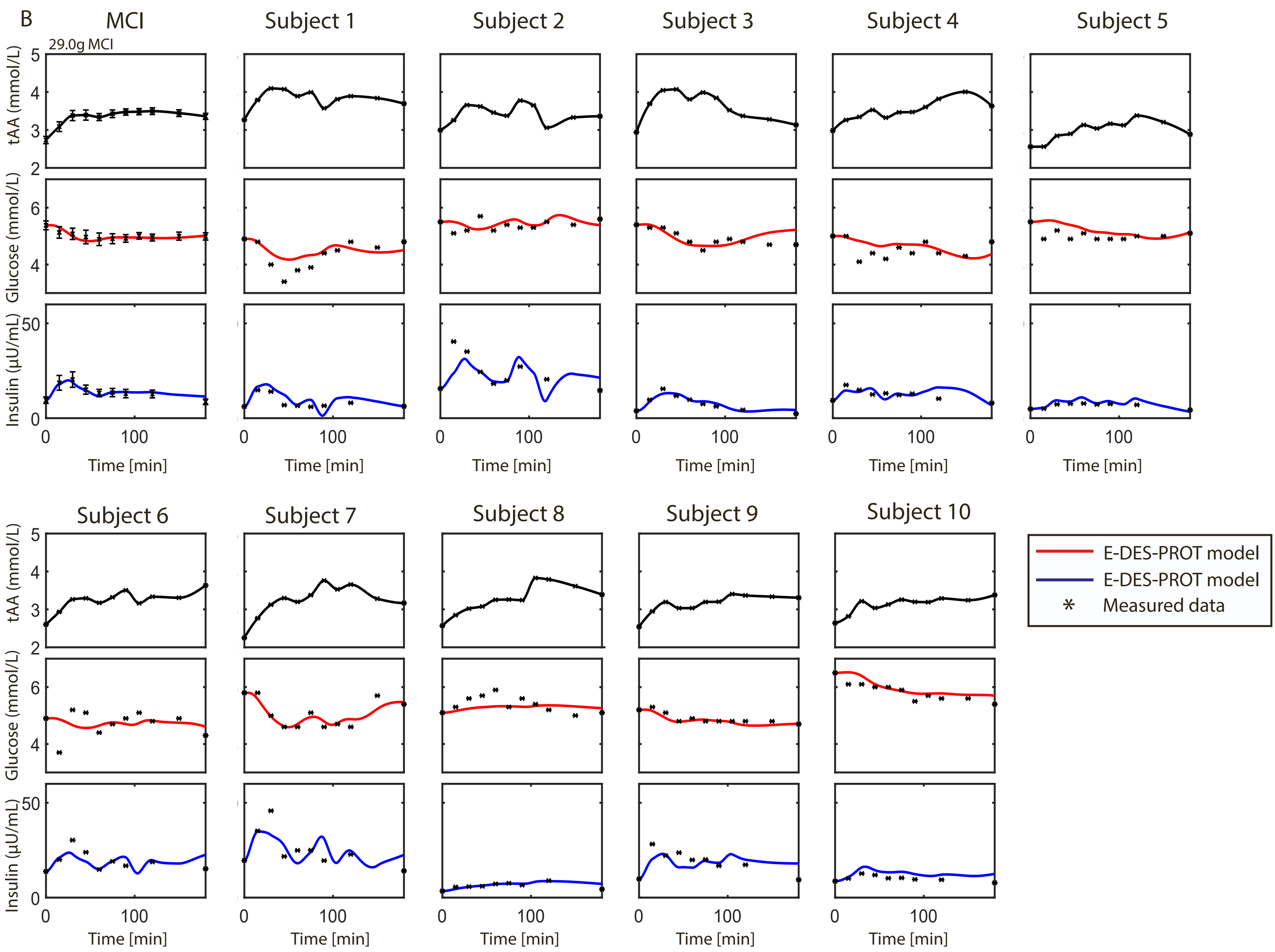

B
MCI
29.0g MCI
Subject 1
Subject 2
Subject 3
Subject 4
Subject 5
Subject 6
Subject 7
Subject 8
Subject 9
Subject 10
tAA (mmol/L)
Glucose (mmol/L)
Insulin (μU/mL)
Time [min]
E-DES-PROT model
E-DES-PROT model
Measured data

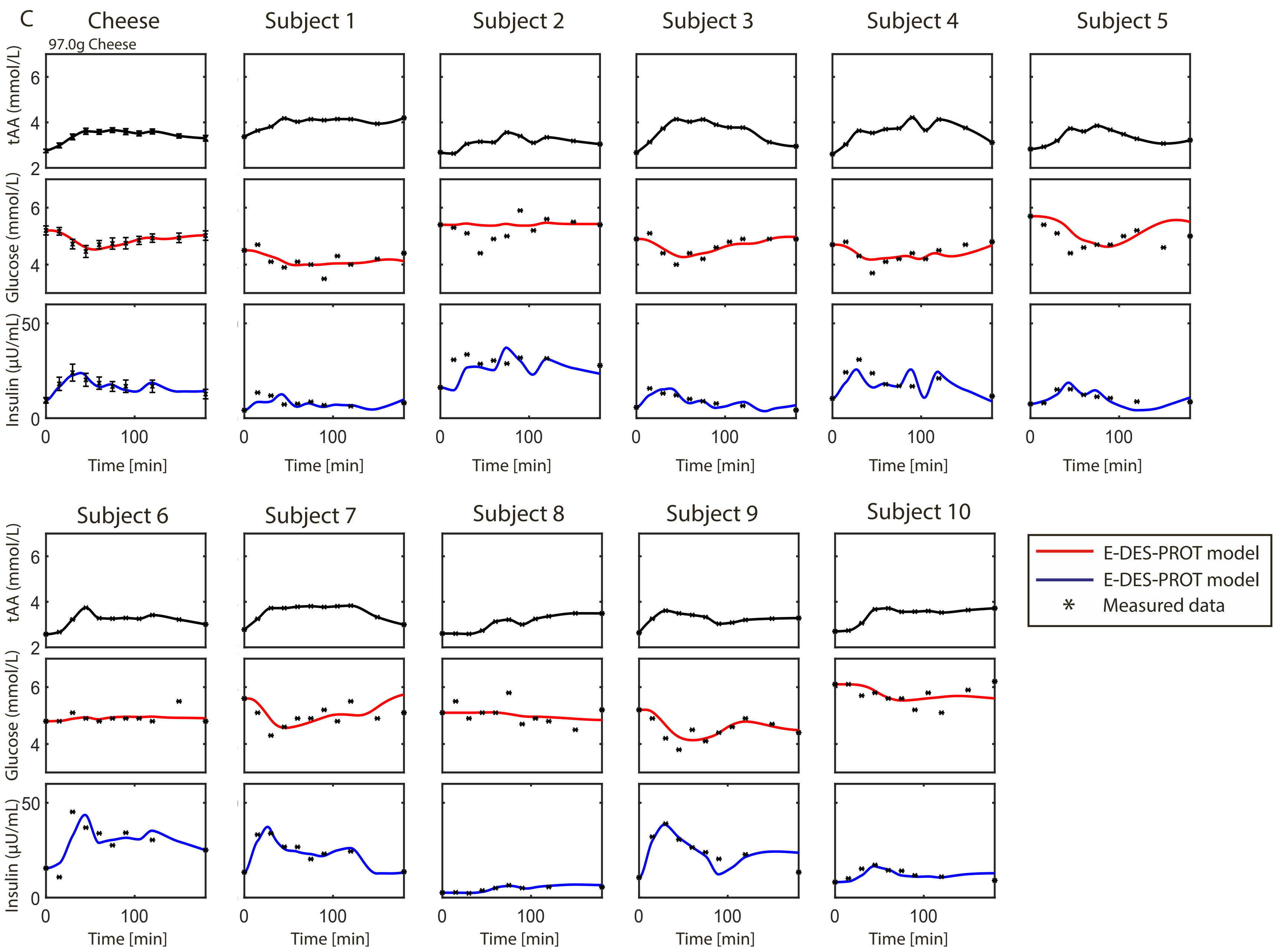
C
Cheese
97.0g Cheese
Subject 1
Subject 2
Subject 3
Subject 4
Subject 5
Subject 6
Subject 7
Subject 8
Subject 9
Subject 10
tAA (mmol/L)
Glucose (mmol/L)
Insulin (μU/mL)
Time [min]
E-DES-PROT model
E-DES-PROT model
Measured data

Data S3: Simulated postprandial responses for all individuals in the randomized single-blind crossover trial study following ingestion of dairy products (LF-UHT, LF-PAS, FF-UHT, and Yoghurt), related to Figure 4.

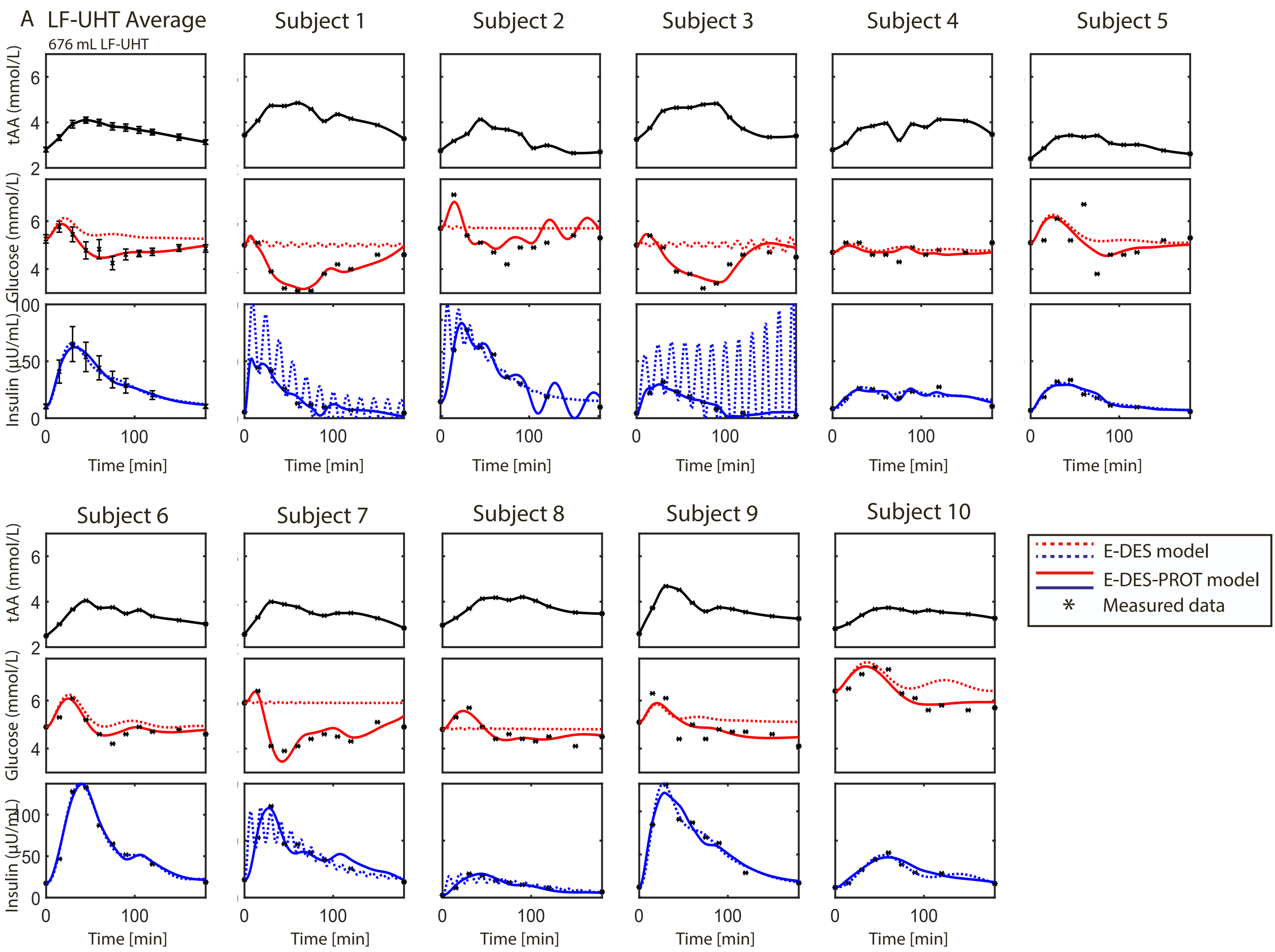
A LF-UHT Average
676 mL LF-UHT
Subject 1
Subject 2
Subject 3
Subject 4
Subject 5
Subject 6
Subject 7
Subject 8
Subject 9
Subject 10
tAA (mmol/L)
Glucose (mmol/L)
Insulin (µU/mL)
Time [min]
E-DES model
E-DES-PROT model
Measured data

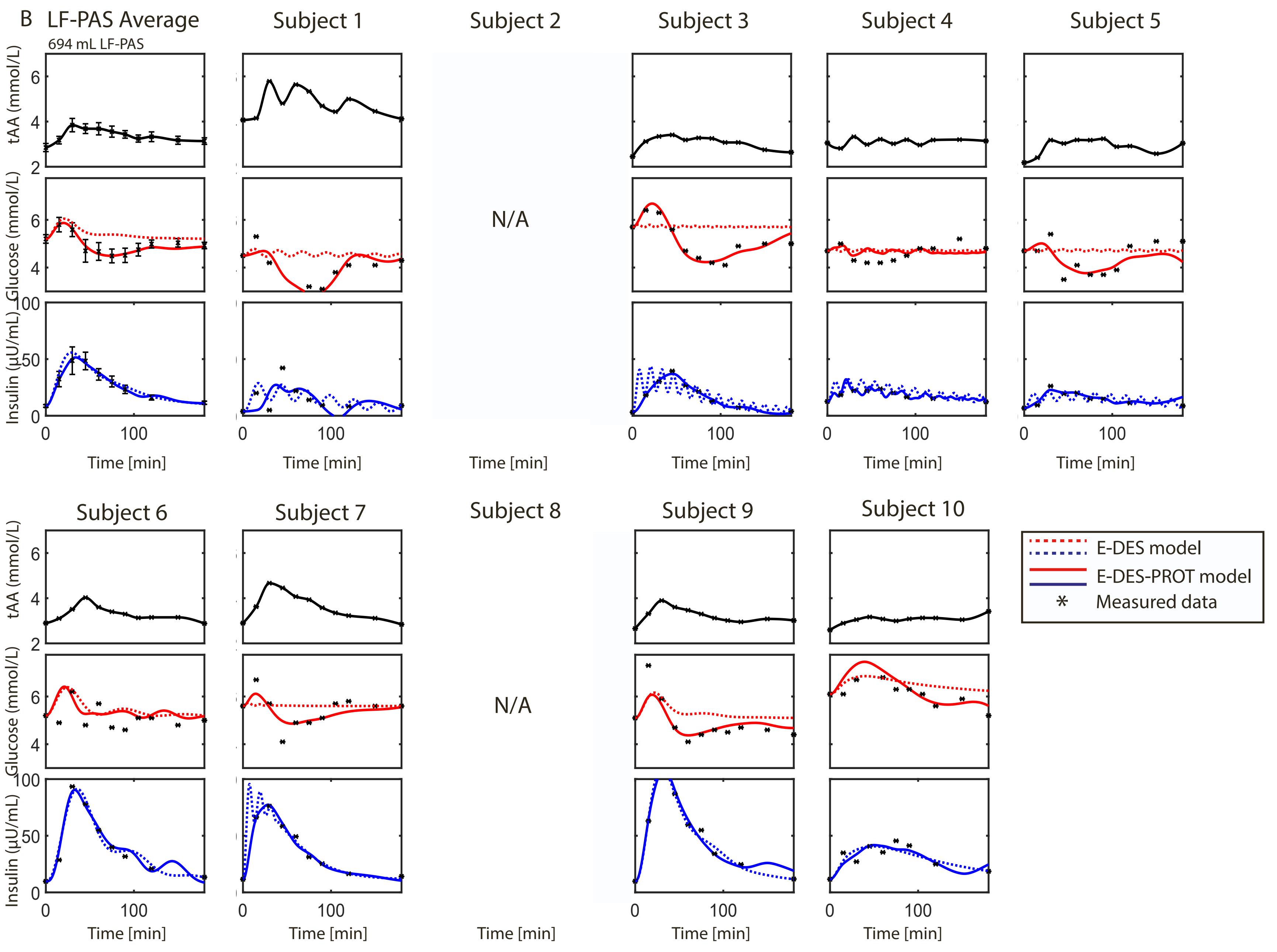
B
LF-PAS Average
694 mL LF-PAS
Subject 1
Subject 2
Subject 3
Subject 4
Subject 5
Subject 6
Subject 7
Subject 8
Subject 9
Subject 10
N/A
N/A
tAA (mmol/L)
Glucose (mmol/L)
Insulin (μU/mL)
Time [min]
E-DES model
E-DES-PROT model
Measured data

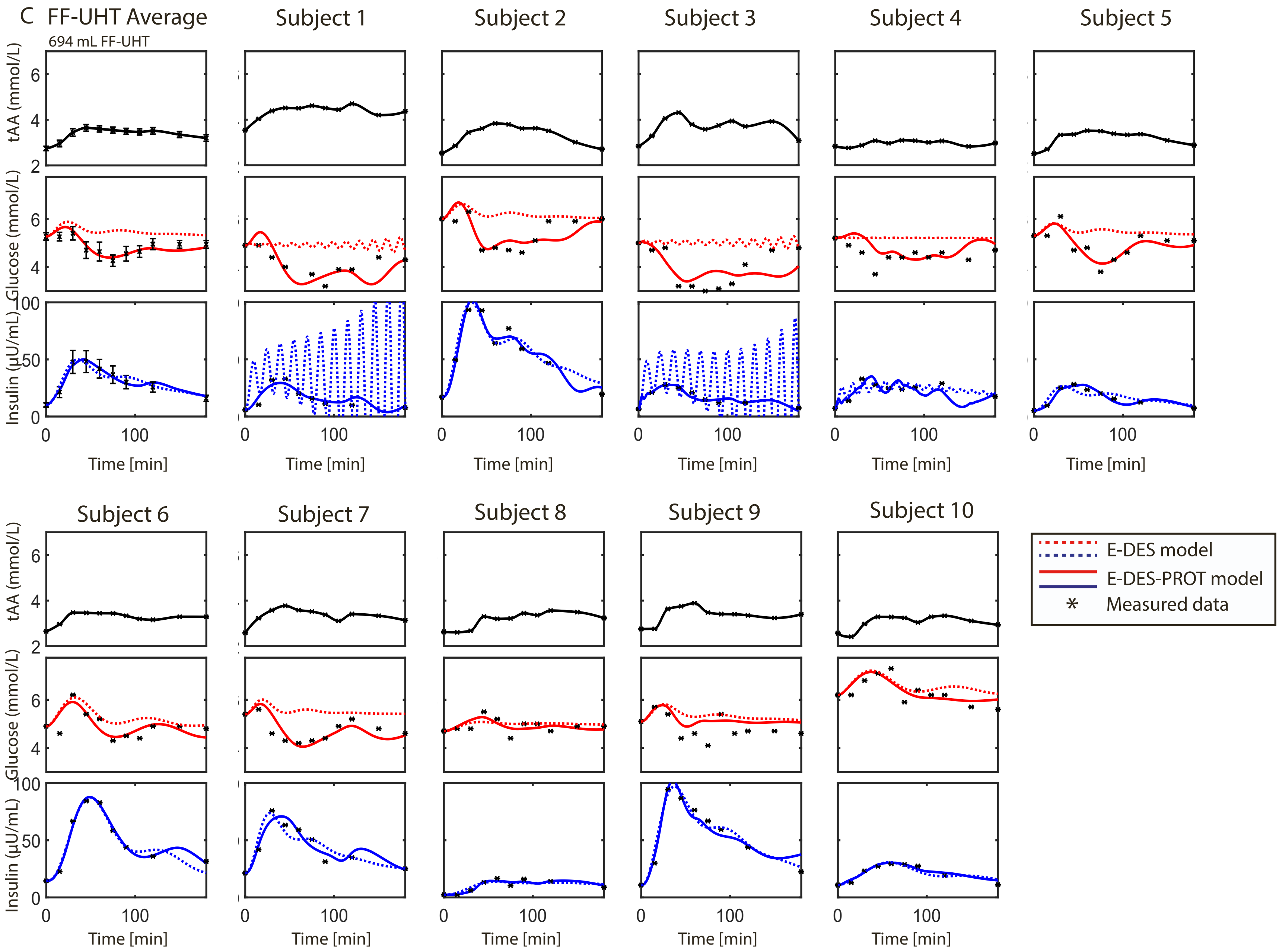
C FF-UHT Average
694 mL FF-UHT
Subject 1
Subject 2
Subject 3
Subject 4
Subject 5
Subject 6
Subject 7
Subject 8
Subject 9
Subject 10
tAA (mmol/L)
Glucose (mmol/L)
Insulin (μU/mL)
Time [min]
E-DES model
E-DES-PROT model
Measured data

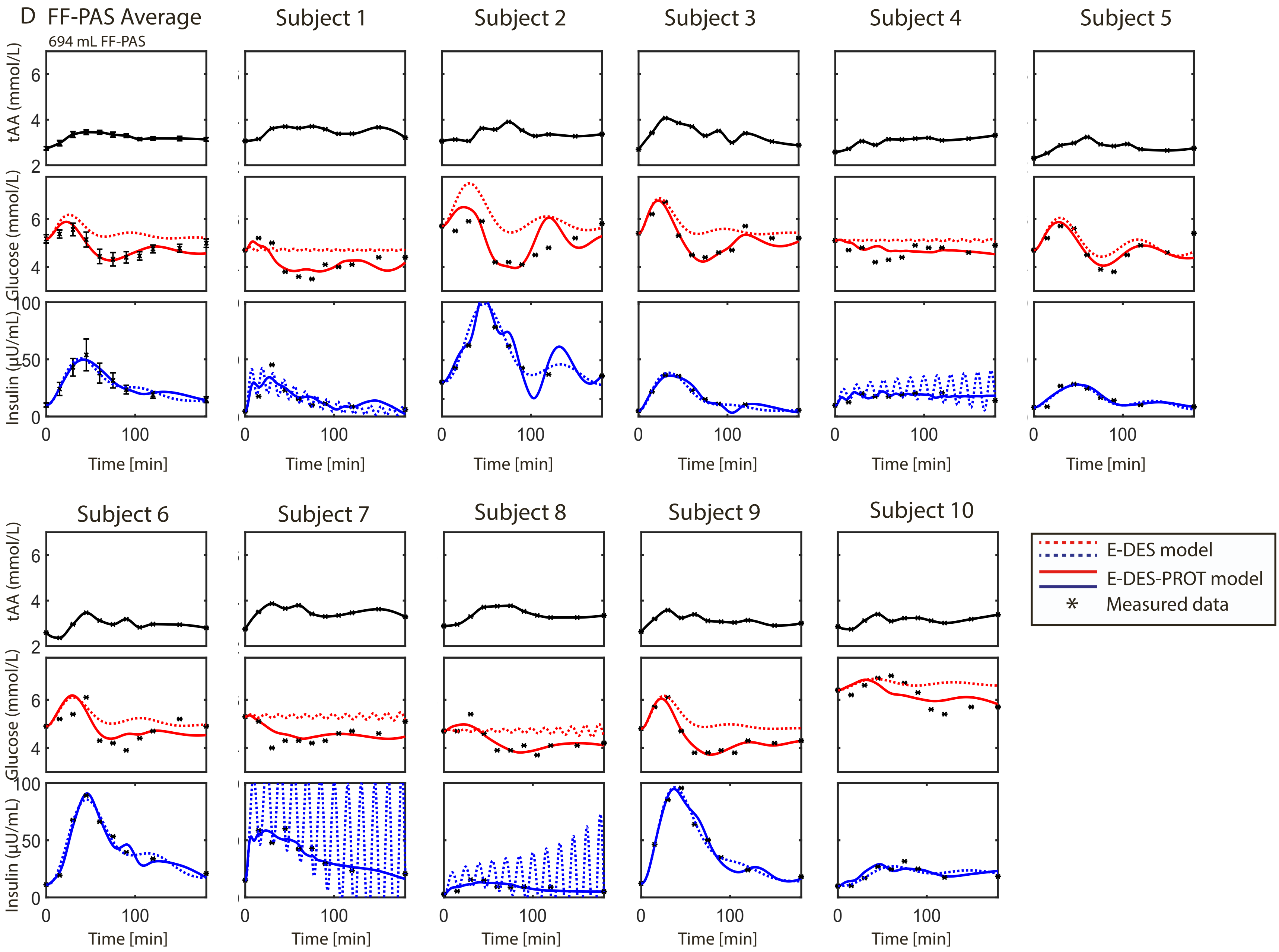

D FF-PAS Average
694 mL FF-PAS
Subject 1
Subject 2
Subject 3
Subject 4
Subject 5
Subject 6
Subject 7
Subject 8
Subject 9
Subject 10
tAA (mmol/L)
Glucose (mmol/L)
Insulin (µU/mL)
Time [min]
E-DES model
E-DES-PROT model
Measured data

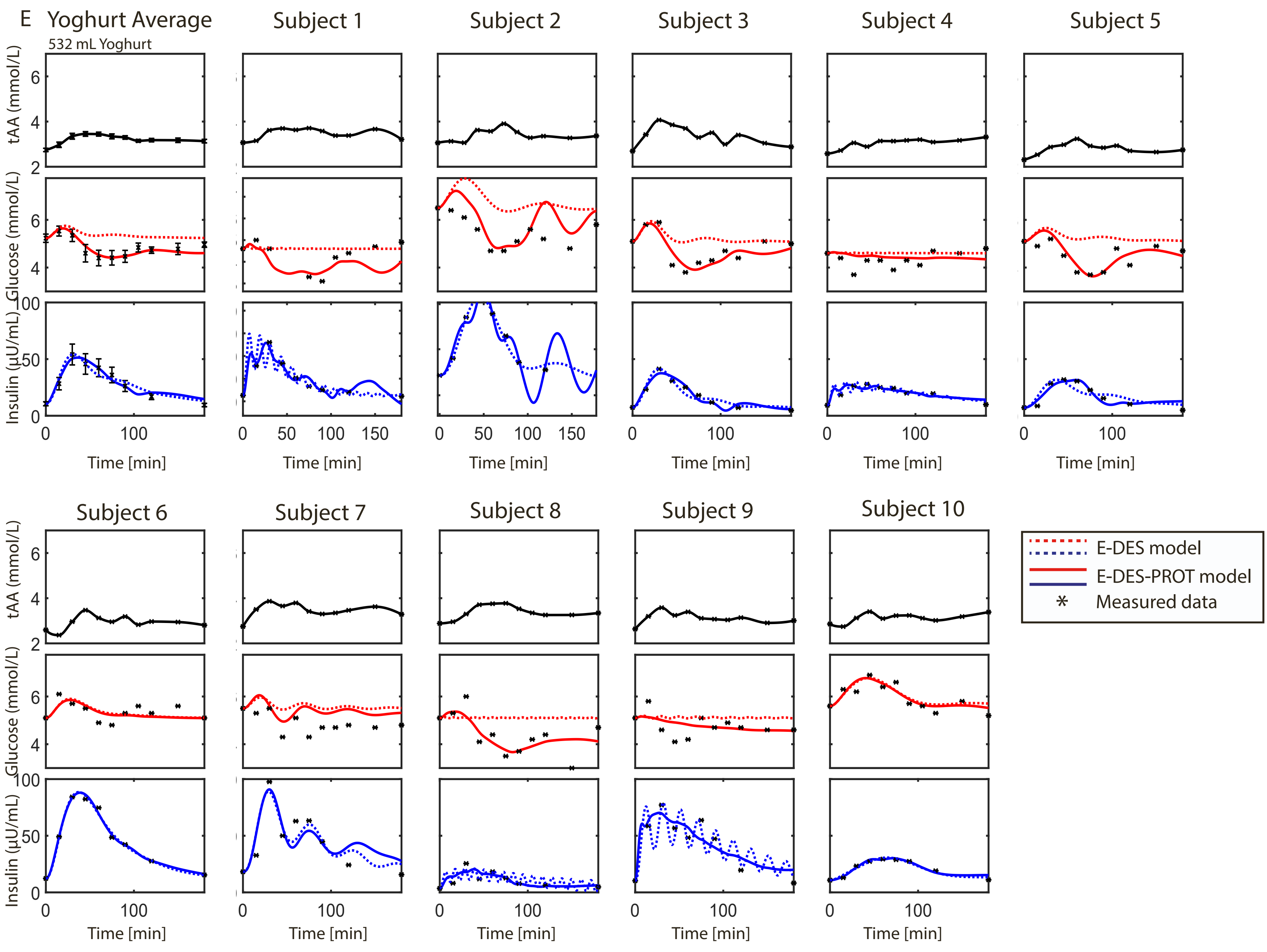

E Yoghurt Average
532 mL Yoghurt
Subject 1
Subject 2
Subject 3
Subject 4
Subject 5
Subject 6
Subject 7
Subject 8
Subject 9
Subject 10
tAA (mmol/L)
Glucose (mmol/L)
Insulin (μU/mL)
Time [min]
E-DES model
E-DES-PROT model
Measured data

Data S4: E-DES-PROT model structure, fluxes, inputs, constants, parameters, related to STAR Methods.

1 E-DES-PROT Model structure

Glucose in the gut

$$\frac{dM_G^{gut}}{dt} = m_G^{meal}(t) - m_G^{pl}(t) \tag{1}$$

$$m_G^{meal}(t) = \sigma k_1^{\sigma} t^{\sigma-1} exp(-(k_1 t)^{\sigma}) D^{meal} \tag{2}$$

$$m_G^{pl}(t) = k_2 M_G^{gut}(t) \tag{3}$$

Glucose in the plasma

$$\frac{dG^{pl}}{dt} = g^{liv}(t) + g^{gut}(t) - g^{non-it}(t) - g^{it}(t) - g^{ren}(t) \tag{4}$$

$$g^{liv}(t) = g_b^{liv} - k_3(G^{pl}(t) - G_b^{pl}) - k_4 \beta I^{if}(t) + k_{11}(AA^{pl}(t) - AA_b^{pl}) \tag{5}$$

$$g^{gut}(t) = \frac{f}{V_G M^b} m_G^{pl}(t) = k_2 \frac{f}{V_G M^b} M_G^{gut}(t) \tag{6}$$

$$g^{non-it}(t) = g_b^{liv} \left(\frac{K_M + G_b^{pl}}{G_b^{pl}} \right) \frac{G^{pl}(t)}{K_M + G^{pl}(t)} \tag{7}$$

$$g^{it}(t) = k_5 \beta I^{if}(t) \frac{G^{pl}(t)}{K_M + G^{pl}(t)} \tag{8}$$

$$g^{ren}(t) = \begin{cases} \frac{c_1}{V_G M^b}(G^{pl}(t) - G_{th}^{pl}), & \text{if } G^{pl}(t) > G_{th}^{pl} \quad (9) \\ 0, & \text{if } G^{pl}(t) \leq G_{th}^{pl} \quad (10) \end{cases}$$

Insulin in the plasma

$$\frac{dI^{pl}}{dt} = i^{pnc}(t) - i^{liv}(t) - i^{if}(t)) \tag{11}$$

$$i^{pnc}(t) = \beta^{-1}(k_6(G^{pl}(t) - G_b^{pl}) + \left(\frac{k_7}{\tau_i}\right) \int (G^{pl}(t) - G_b^{pl})dt + \left(\frac{k_7}{\tau_i}\right) G_b^{pl} + (k_8 \tau_d)\frac{dG^{pl}}{dt} + k_{12}\frac{dAA^{pl}}{dt} + k_{13}(AA^{pl}(t) - AA_b^{pl})) \tag{12}$$

$$i^{liv}(t) = k_7 \frac{G_b^{pl}}{\beta \tau_i I_b^{pl}} I^{pl}(t) \tag{13}$$

$$i^{if}(t) = k_9(I^{pl}(t) - I_b^{pl}) \tag{14}$$

Insulin in the interstitial fluid

$$\frac{dI^{if}}{dt} = i^{if}(t) - i^{it}(t) \tag{15}$$

$$i^{it}(t) = k_{10}(I^{if}(t)) \tag{16}$$

2 E-DES-PROT model inputs, fluxes and constants

Table S5: Overview of the E-DES-PROT model (input)variables

Name	Description	Units
t	Time	min
$M_G^{gut}(t)$	Glucose mass in the gut	mg
$G^{pl}(t)$	Plasma glucose concentration	mmol/L
$I^{pl}(t)$	Plasma insulin concentration	mU/L
$AA^{pl}(t)$	Plasma amino acid concentration	μmol/L
$I^{if}(t)$	Interstitial fluid insulin concentration	mU/L
D^{meal}	Glucose intake	mg
M^b	Body mass	kg

Table S6: Overview of the E-DES-PROT model fluxes

Name	Description	Units
$m_G^{meal}(t)$	Glucose mass entering from stomach	mg/min
$m_G^{pl}(t)$	Glucose mass leaving to plasma	mg/min
$g^{liv}(t)$	Glucose production by the liver (EGP)	mmol/L/min
$g^{gut}(t)$	Glucose entering from the gut	mmol/L/min
$g^{non-it}(t)$	Glucose uptake by insulin-independent tissue	mmol/L/min
$g^{it}(t)$	Glucose uptake by insulin-dependent tissue	mmol/L/min
$g^{ren}(t)$	Renal glucose elimination	mmol/L/min
$i^{pnc}(t)$	Pancreas insulin secretion	mU/L/min
$i^{if}(t)$	Insulin flowing into interstitial fluid	mU/L/min
$i^{liv}(t)$	Insulin uptake by the liver	mU/L/min
$i^{it}(t)$	Insulin usage by insulin dependent tissue	mU/L/min

Table S7: Overview of the E-DES-PROT model constants

Name	Description	Units	Value
G_b^{pl}	Basal plasma glucose	mmol/L	$G^{pl}(0)$
I_b^{pl}	Basal plasma insulin	mU/L	$I^{pl}(0)$
AA_b^{pl}	Basal plasma amino acid	μmol/L	$AA^{pl}(0)$
g_b^{liv}	Basal endogenous glucose production	mmol/L/min	0.043
G_{th}^{pl}	Renal threshold	mmol/L	9
V_G	Glucose distribution volume in plasma	L/kg	17/70
β	Unit conversion factor	(mmol/L)/(mU/L)	1
f	Unit conversion factor from mmol to mg glucose	mmol/mg	0.005551
τ_i	Integral time constant	min	31
τ_d	Derivative time constant	min	3
$c1$	Rate constant of glomerular filtration	1/min	0.1

3 E-DES-PROT parameter values

Table S8: Parameter values for reference simulation: Average LF-UHT

Name	Description	Units	Value
$k1$	Rate constant of glucose appearance in the gut	1/min	0.0155
$k2$	Rate constant of gut emptying	1/min	0.28
$k3$	Rate constant of ΔG suppression of EGP when $G^{pl} > G_b^{pl}$	1/min	6.07e-3
$k4$	Rate constant of I^{if}-dependent suppression of EGP	1/min	2.35e-4
$k5$	Rate constant of insulin-dependent glucose uptake	1/min	0.087
$k6$	Rate constant of ΔG dependent insulin production	1/min	3.01
$k7$	Rate constant of $\int$G dependent insulin production	1/min	1.15
$k8$	Rate constant of dG/dt dependent insulin production	1/min	10.99
$k9$	Rate constant of insulin outflow from plasma to interstitial fluid	1/min	3.83e-2
$k10$	Rate constant of interstitial fluid insulin utilization	1/min	0.28
$k11$	Rate constant of ΔAA increase of EGP when $AA^{pl} > AA_b^{pl}$	1/min	1.25e-7
$k12$	Rate constant of dAA/dt dependent insulin production	1/min	1.85e-3
$k13$	Rate constant of ΔAA dependent insulin production	1/min	3.40e-2
σ	Shape factor of the gastric emptying pattern	dimensionless	1.34
Km	Michaelis-Menten constant for glucose uptake	mg/dL	13.2
