# Supplementary figures and images for: E-DES-PROT: A novel computational model to describe the effects of amino acids and protein on postprandial glucose and insulin dynamics in humans
Source: iScience. 2023 Feb 18;26(3):106218. doi: 10.1016/j.isci.2023.106218 (PMC9989689; doi:10.1016/j.isci.2023.106218)

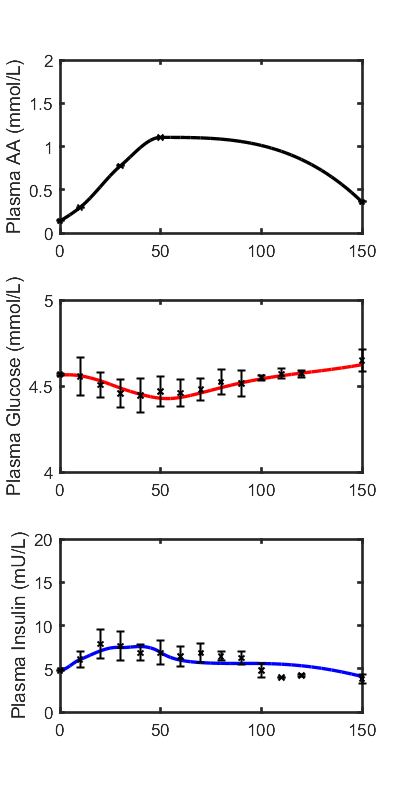

Supplement: Data S1. MATLAB implementation of the E-DES-PROT model used in the manuscript, related to STAR Methods [file mmc2.zip › Data S1/Example_figure.png]
